# Best practices and benchmarks for intact protein analysis for top-down mass spectrometry

**DOI:** 10.1038/s41592-019-0457-0

**Published:** 2019-06-27

**Authors:** Daniel P. Donnelly, Catherine M. Rawlins, Caroline J. DeHart, Luca Fornelli, Luis F. Schachner, Ziqing Lin, Jennifer L. Lippens, Krishna C. Aluri, Richa Sarin, Bifan Chen, Carter Lantz, Wonhyeuk Jung, Kendall R. Johnson, Antonius Koller, Jeremy J. Wolff, Iain D. G. Campuzano, Jared R. Auclair, Alexander R. Ivanov, Julian P. Whitelegge, Ljiljana Paša-Tolić, Julia Chamot-Rooke, Paul O. Danis, Lloyd M. Smith, Yury O. Tsybin, Joseph A. Loo, Ying Ge, Neil L. Kelleher, Jeffrey N. Agar

**Affiliations:** 1 Barnett Institute of Chemical and Biological Analysis and Departments of Chemistry & Chemical Biology and Pharmaceutical Sciences, Northeastern University, Boston, MA USA; 2 Departments of Chemistry and Molecular Biosciences and the Proteomics Center of Excellence, Northwestern University, Evanston, IL USA; 3 Department of Cell and Regenerative Biology, Department of Chemistry, Human Proteomics Program, University of Wisconsin-Madison, Madison, WI USA; 4 Amgen Research, Discovery Attribute Sciences, Amgen, Thousand Oaks, CA USA; 5 Alnylam Pharmaceuticals, Cambridge, MA USA; 6 Biogen, Cambridge, MA USA; 7 Department of Chemistry and Biochemistry, Department of Biological Chemistry, and UCLA/DOE Institute of Genomics and Proteomics, University of California, Los Angeles, Los Angeles, CA USA; 8 Bruker Daltonics, Billerica, MA USA; 9 Biopharmaceutical Analysis Training Laboratory, Northeastern University, Burlington, MA USA; 10 The Pasarow Mass Spectrometry Laboratory, The Jane and Terry Semel Institute for Neuroscience and Human Behavior, David Geffen School of Medicine, University of California, Los Angeles, Los Angeles, CA USA; 11 Environmental Molecular Sciences Laboratory, Pacific Northwest National Laboratory, Richland, WA USA; 12 Mass Spectrometry for Biology Unit, Institut Pasteur, USR 2000, CNRS, Paris, France; 13 Eastwoods Consulting, Boylston, MA USA; 14 Department of Chemistry, Genome Center of Wisconsin, University of Wisconsin-Madison, Madison, WI USA; 15 Spectroswiss, Lausanne, Switzerland

**Keywords:** Proteomics, Proteomic analysis, Proteins, Mass spectrometry

## Abstract

One gene can give rise to many functionally distinct proteoforms, each of which has a characteristic molecular mass. Top-down mass spectrometry enables the analysis of intact proteins and proteoforms. Here members of the Consortium for Top-Down Proteomics provide a decision tree that guides researchers to robust protocols for mass analysis of intact proteins (antibodies, membrane proteins and others) from mixtures of varying complexity. We also present cross-platform analytical benchmarks using a protein standard sample, to allow users to gauge their proficiency.

## Abstract (web summary)

The Consortium for Top-Down Proteomics presents a decision-tree-based guide to sample preparation and analysis protocols for researchers performing top-down mass-spectrometry-based analysis of intact proteins.

## Main

Mutations, polymorphisms, RNA processing and post-translational modifications (PTMs) such as acetylation, methylation and phosphorylation can lead to a single gene producing many functionally distinct ‘proteoforms’^1^. These proteoforms can have different effects on important biological processes, including gene regulation, cell signaling and protein activity; consequently, the ability to characterize these species is essential for an understanding of the biological response to disease. The identity of a proteoform can often be inferred^2^ from an accurate experimentally determined intact mass^3^. One can increase the sensitivity of intact-mass-based proteoform identification by determining the relative abundance of a particular amino acid by using isotopic labeling, by using mass similarities to cluster proteoforms into gene families and by reducing the search space using sample-specific search databases^2^. Localizing PTMs, and in some cases the definitive proteoform identifier, requires tandem mass spectrometry (MS^n^) analysis. The measurement of intact protein mass followed by MS^n^ has been coined ‘top-down’ mass spectrometry^4–8^, with its origins in Fenn and colleagues’ discovery that large biomolecules could be ionized^9^ and fragmented^10–12^ using electrospray ionization (ESI)-MS. Top-down MS protocols, unlike widely used bottom-up protocols^13,14^, do not require endoproteinase digestion before analysis, do not conflate proteoforms and tend to complement native MS analysis.

One advantage of ESI over the alternative ‘soft’ ionization method, matrix-assisted laser desorption-ionization (MALDI), is that ESI imparts more charge per protein. This enables the mass determination of large biomolecules using mass analyzers with moderate mass-to-charge ratio upper limits (for example, *m/z* ≤ 4,000), which happen to offer the highest resolving power. Higher charge per molecular mass also facilitates gas-phase fragmentation and, therefore, the characterization of primary sequence and PTMs by MS^n^ (refs. ^15,16^). Because of this superior fragmentation and the ability to interface with liquid chromatography (LC) systems, ESI is used for most top-down MS experiments. Projects requiring rapid MS analysis^17^, the ability to analyze hundreds of proteins in a single spectrum, protein imaging capabilities, or less signal suppression by common protein buffer components^18^ may be better suited for MALDI-MS.

Compared to bottom-up workflows, top-down approaches provide additional layers of information, including detecting modifications that are removed or scrambled^19^ during peptide sample preparation (for example, *S*-thiolation), elucidating functional relationships (for example, cross-talk) between PTMs on the same protein molecule, characterizing drug–target interactions, observing important modifications on biopharmaceuticals, and identifying and quantifying distinct proteoforms that would have been convoluted by endoproteinase digestion^20–24^. In addition, sample preparation for intact protein MS comprises fewer steps than bottom-up approaches and does not require chemical modification (for example, reduction and alkylation), thereby reducing the number of experimental artifacts^25^. Current top-down sample cleanup methods (for example, protein precipitation^26^ and molecular weight cut-off (MWCO) ultrafiltration) are not applicable to all sample types or downstream MS analyses. The demand for robust, generally applicable methods for intact protein MS is the most common request made to members of the Consortium for Top-Down Proteomics^27,28^ (http://topdownproteomics.org/).

Our goal here is to address this unmet need, by providing a guide to enable users with all levels of expertise to acquire high-quality intact protein mass spectra by ESI-MS. First we discuss signal suppression associated with common buffer components and biotherapeutic excipients. This provides the rationale for most failed intact MS measurements and, in addition, a path to designing MS-compatible buffers. Then, we present a decision tree based on sample composition and experimental goals, which guides users to a best-practices protocol and corresponding benchmark data.

## Origins of signal suppression and signal spreading

Biological, biochemical and biotherapeutic sample preparations usually contain numerous interfering substances (for example, salts, detergents, chaotropes and buffers) that lead to signal suppression during ESI-MS analysis. To provide a theoretical context, we describe the two major drivers of the quality of intact protein (positive ion) ESI-MS and how these are affected by interfering substances. The first driver of quality is the formation of desolvated protein ions, which can be understood in terms of a few critical steps during the ESI process^16,29,30^. Interfering substances generally affect the ESI process after the formation of nanodroplets at the Rayleigh charge limit. Two salient, often opposing, processes that occur within these nanodroplets are the partitioning of net charge toward the droplet surface and the minimization of solvation energy. Polar species such as salts and native proteins partition toward the droplet interior to optimize solvation energy; their ionization, therefore, requires evaporation of solvent molecules^16^. Hydrophobic species such as detergent monomers and unfolded proteins migrate to the droplet surface to optimize solvation energy and, in a faster process that requires less energy, evaporate or are ejected. Many of the techniques presented here for reducing signal suppression can be rationalized within the framework above. For example, organic solvents that decrease surface tension should promote the ionization of both polar and nonpolar analytes; detergents partition to the surface, where they can outcompete analytes for a limited number of protons; organic solvents and acids that unfold proteins should promote ejection-based ionization; native MS (nMS) requires greater desolvation energy and is more sensitive to polar contaminants.

The second driver of the quality of intact protein MS is signal spreading (that is, the distribution of the signal from a single proteoform across multiple channels), which increases with protein mass. Each channel has its own respective noise; consequently, the cumulative noise increases proportionally to the number of channels. The ESI process promotes signal spreading, via adduct formation, by increasing the concentrations of interfering substances and proteins. Heavy isotopes and charge states further distribute signal intensity across multiple channels; the former can be mitigated by isotope depletion^31^. Here we describe experimental techniques that minimize signal spreading (increase signal-to-noise ratio, or S/N), including using nMS to reduce the number of charge states, and the use of volatile salts (for example, ammonium acetate) or purification to minimize the effects of alkaline salts.

## Signal suppression by common buffer components

**Fig. 1:**
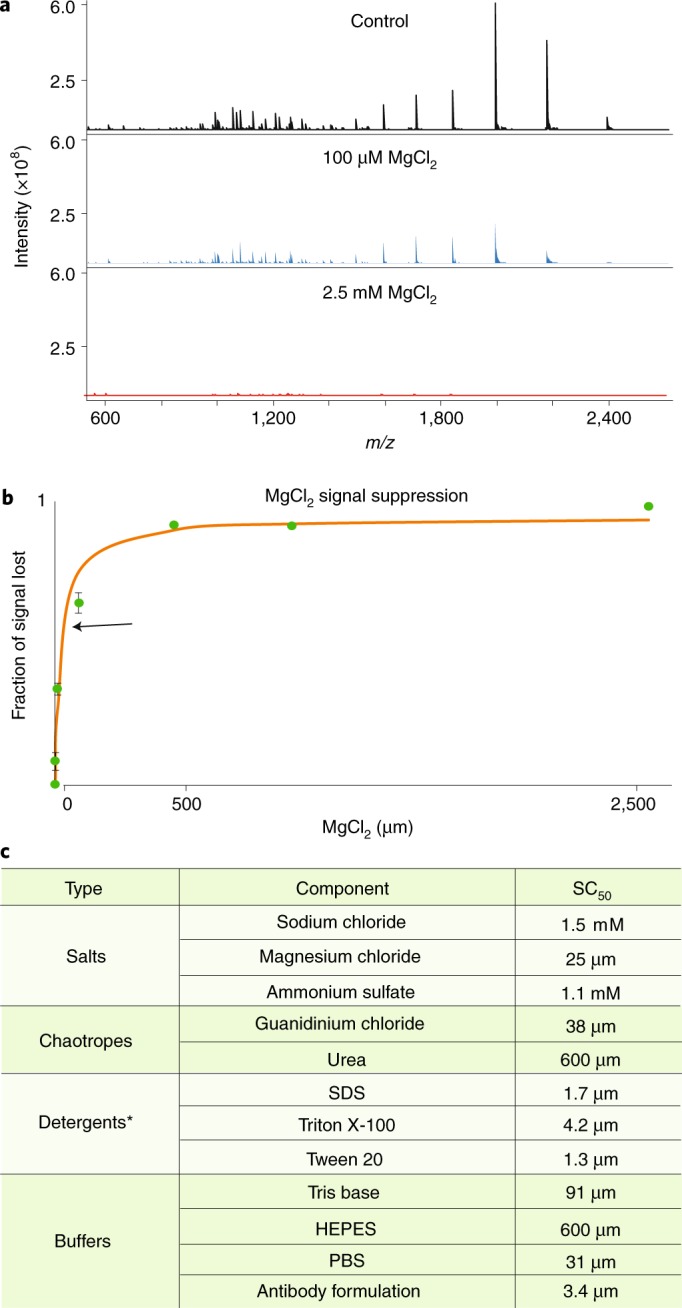
Common buffer components suppress MS signal. **a**, MgCl_2_ reduces signal (and S/N) in a concentration-dependent manner. **b**, Fit of experimental data to determine the concentration of MgCl_2_ required for 50% signal suppression (SC_50_; black arrow), **c**, Table of common buffer components and the concentration threshold for 50% SC_50_ (experimental data curves and their fits are shown in Supplementary Fig. 1) and calculations in Supplementary Note 2. Detergents compatible with mass spectrometry are discussed in Protocol 2b. *Signal suppression by detergents is less pronounced above their critical micellar concentration (CMC) (described in Protocol 2b).

Using the intact protein standard mixture (ubiquitin, myoglobin, trypsinogen and carbonic anhydrase) established by the National Resource for Translational and Developmental Proteomics (NRTDP) (http://nrtdp.northwestern.edu/protocols/), we evaluated common buffer components (Fig. 1) to quantify the concentration required for 50% signal suppression during direct infusion ESI. By analogy to half-maximum inhibitory concentration (IC_50_) nomenclature, we termed this metric the half-maximum suppression concentration (SC_50_) (Fig. 1, Supplementary Fig. 1). At their typical concentrations, all common buffer additives suppressed ESI signal considerably. Consistent with the mechanisms of ESI ionization described above, detergents produced the most signal suppression, less volatile (for example, metallic) salts produced intermediate suppression and volatile components lowest suppression. Additional details of the experimental parameters used here are provided in Supplementary Notes 1 and 2.

The SC_50_ values given in Fig. 1 allow users to design MS-compatible buffers. In addition, the SC_50_ and buffer composition serve as the entry point into the decision tree outlined below, leading users to the appropriate protocol. Although the trends in SC_50_ values reported here should generally be consistent across MS platforms, parameter-dependent variations in the reported values are likely (in particular, flow rate, voltages, temperatures, and pressures that affect ionization and desolvation). Here, for example, we calculated SC_50_ obtained by direct infusion using a standard microflow ESI source (about a microliter per minute), but nano-ESI (less than a microliter per minute) is less affected by salts because of the order-of-magnitude decrease in initial droplet size^32,33^.

## Intact protein MS (IPMS) decision tree

**Fig. 2:**
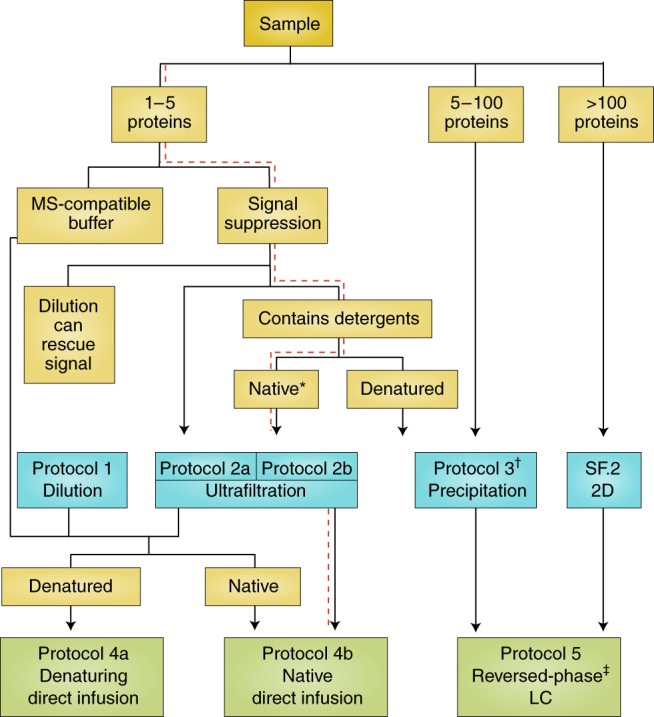
Decision tree for intact protein sample clean-up, preparation and analysis. The red dashed line, for example, denotes the decision path for the native MS analysis of a membrane protein. *LC can also be applied at this stage in the decision tree. ^†^Minimally complex protein samples prepared following Protocol 3 can be analyzed via denaturing direct infusion (Protocol 4a) if desired. Supplementary Fig. 2 (SF.2) shows a recommended example of 2D separation following the GELFrEE protocol. ^‡^Other viable alternative separation techniques include capillary zone electrophoresis, ion exchange and size exclusion chromatography.

The IPMS decision tree (Fig. 2) directs practitioners to a protocol or a combination of protocols based on buffer composition, the number of proteins in the sample, and whether native or denaturing conditions are to be used. Consider, for example, a purified protein in phosphate-buffered saline (PBS). Based on the 1.5 mM SC_50_ exhibited by NaCl (Fig. 1c) and the 137 mM NaCl present in PBS, a protein sample in PBS requires a 91-fold dilution to achieve 50% of the potential MS signal. Therefore, if the protein concentration is greater than 90 µM and salt adducts will not impede data analysis, the sample can be diluted following Protocol 1. Otherwise, sample cleanup by ultrafiltration using spin cartridges with a MWCO-membrane is recommended following Protocol 2.

Interest in certain PTMs (for example, metallation) or protein complex quaternary structure would dictate the use of native MS methods following Protocol 4b; otherwise the denaturing MS Protocol 4a is recommended. Depending on the complexity of the sample, additional separation techniques such as GELFrEE may be required (Supplementary Fig. 2). The objective of this decision tree is to provide a proven workflow for any sample, not to rule out alternative methods. For example, depending on sample stability, user expertise and available resources, precipitation (Protocol 3), size exclusion ‘spin cartridges’, or LC (Protocol 5) could be suitable alternatives to MWCO ultrafiltration. All protocols and benchmarks referenced by the decision tree and alternative methods are summarized below and further detailed in Supplementary Notes 1−5 and Supplementary Protocols 1−5.

## Protein standards and benchmarks

To promote standardization and allow users to benchmark their own data using readily available proteins, we provided representative results for each protocol using the following commercially available standards: (i) the NRTDP intact protein standard mixture (see Supplementary Note 1 for preparation instructions), (ii) NIST monoclonal antibody reference material 8671 (NISTmAb), containing humanized IgG1ĸ in 12.5 mM L-histidine, 12.5 mM L-histidine HCl (pH 6.0), and (iii) Sigma bacteriorhodopsin from *Halobacterium salinarum* (B0184). Benchmarks for mass accuracy depend upon the instrumentation platform and have been reviewed^3,34–39^. Rules of thumb include requiring 10 p.p.m. accuracy for modern Fourier transform MS and 20 p.p.m. accuracy for modern quadrupole time-of-flight (QTOF) MS. We suggest the use of ProForma notation^40^ for standardized proteoform nomenclature, and note that the PeptideMass tool (https://web.expasy.org/peptide_mass/) can be used to calculate the mass of a given sequence or of proteoforms contained in the UniProt database.

## Protocol 1: sample preparation by dilution of interfering substances

**Fig. 3:**
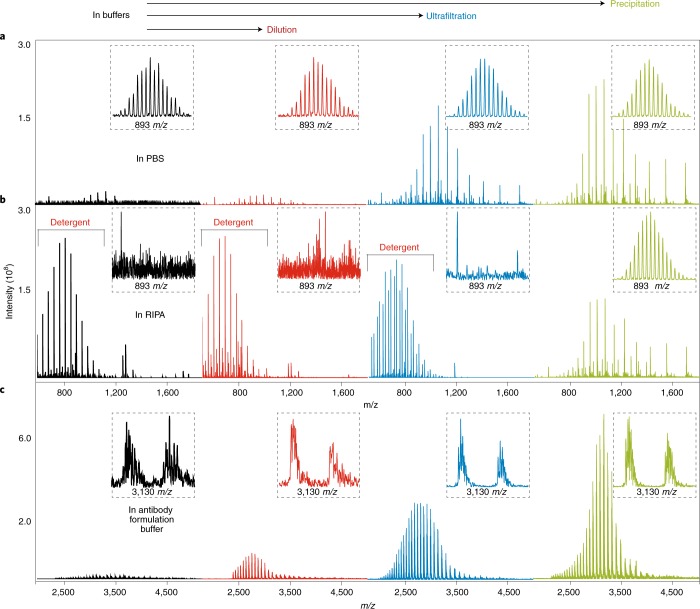
Dilution (Protocol 1), MWCO ultrafiltration (Protocol 2a) and precipitation (Protocol 3) sample preparation protocols applied to common buffers. **a**,**b**, Protein standard mixture in PBS (**a**) and detergent-containing RIPA buffer (**b**). In the buffer containing harsh detergents, protein signal is attained only with precipitation. **c**, NISTmAb in 12.5 mM L-histidine, 12.5 mM L-histidine HCl (pH 6.0). Mass spectra were obtained using a Fourier transform ion cyclotron resonance MS (FT-ICR) (Bruker Daltronics SolariX 9.4T MS) using denaturing direct infusion (Protocol 4a). See Supplementary Fig. 3 for additional results with ‘gentle elution’ immunoaffinity elution buffer and a second antibody buffer.

Consistent with the mechanisms of ESI and signal spreading detailed above, common buffer components render proteins undetectable by MS (Fig. 3). Minimally complex, concentrated protein solutions can often be analyzed by direct infusion, following dilution to ~1 µM final protein concentration in the appropriate sample buffer. Users should consider using this protocol if dilution can decrease the concentration of a given interfering substance below its SC_50_ value (Fig. 1, Supplementary Protocol 1). Assuming a practical upper limit of ~10 mM protein concentration, this protocol is potentially applicable to any of the components listed in Fig. 1. As detailed above, however, nMS utilizes an ESI process that is more sensitive to many interferents, including salts. Consequently, dilution is less likely to adequately improve nMS. Protocol 4 describes methods to dilute native proteins into whichever solution will be used to introduce samples to the MS. However, mass spectra obtained by this method have the lowest S/N of any of the protocols described here and may contain adducts.

## Protocol 2: sample preparation using MWCO ultrafiltration

We recommend remediating nonvolatile salt adducts by buffer exchange into a solution of volatile salts. The MWCO of the ultrafiltration device should not exceed half the molecular mass of any given protein in a sample to prevent possible sample loss. No particular pH is optimal for all proteins, but pH extremes should be avoided, as should pH that is equivalent to a protein’s pI, where protein solubility is at a local minimum^41^. We recommend using ammonium acetate throughout these protocols owing to its volatility and ability to act as a stabilizing background electrolyte during ESI^42^. Ammonium acetate provides maximal buffering around pH 4.75 (acetate) and 9.25 (ammonium), and results in a neutral pH upon dissolving in water (approximate pH 6.5−7). Before adding protein sample, MWCO-ultrafiltration devices should be rinsed with the appropriate buffer. Additional details for this method can be found in Supplementary Protocol 2.

### Protocol 2a, soluble proteins

On the basis of the protein masses in the NRTDP intact protein standard, we recommend using a MWCO of 3 kDa according to the manufacturer’s instructions. The protein preparation should be subjected to three (1:20 dilution) buffer exchanges into 10 mM ammonium acetate (pH 6.5) using a MWCO-ultrafiltration device, followed by an additional three exchanges into 2.5 mM ammonium acetate (pH 6.5) (exemplary data in Fig. 3, Supplementary Figs. 3 and 4, see also Supplementary Protocol 2a and Supplementary Note 3). Denaturing and nondenaturing samples can then be diluted and introduced to the MS as described below in Protocol 4a.

### Protocol 2b, native membrane proteins

Membrane proteins are estimated to account for 23% of the total human proteome and represent ~60% of targets for currently approved drugs^43,44^. The mass analysis of native, intact membrane proteins can further provide key information regarding stoichiometry, ligand binding and lipid association. A typical analysis of a membrane protein complex requires either size-exclusion chromatography (SEC) or MWCO ultrafiltration to remove alkali salt adducts while maintaining the detergent used to solubilize the protein (Supplementary Protocol 2b)^45^. This differs fundamentally from the MWCO ultrafiltration used during filter-aided sample preparation (FASP) to improve the bottom-up proteomics analysis of membrane proteins, which removes detergents^46,47^. For users interested in native membrane proteins, we recommend the protocols of Robinson and coworkers^45^. Their protocols are based on comprehensive optimization and include a complete list of non-ionic detergents compatible with MS and detailed sample preparation considerations. We demonstrate an example application of Robinson and coworkers’ protocols for the native tetramer of Aquaporin Z (AqpZ) from *Escherichia coli* (Supplementary Fig. 5).

## Protocol 3: sample preparation using protein precipitation

Common precipitation protocols use organic solvents to agglomerate proteins while leaving small molecules, including salts and detergents, solubilized. Whereas MWCO ultrafiltration using Protocol 2a does not rescue protein signal from a preparation containing harsh surfactants (for example, SDS and Triton), precipitation of proteins following Protocol 3 does (Fig. 3, Supplementary Protocol 3). A volume ratio of 1:1:4:3 of aqueous protein sample:chloroform:methanol:water is recommended to precipitate proteins^26^. The supernatant is removed by aspiration, and the precipitated pellet can be further washed with one more addition and removal of methanol. Pellets are resolubilized for 15 minutes at −20 °C using a small volume of 80% (v/v) formic acid (~25% of the starting volume) and are then diluted to the starting sample volume with HPLC-grade water or a solution of volatile salts (for example, ammonium acetate)^48^. As an alternative method, acetone precipitation has the distinct advantage of leaving many proteins folded. This method, however, has been shown to modify proteins with +98 Da adducts^49^, requires longer incubation at −20 °C (at least 1 h), requires that all steps be performed at or below 0 °C to maximize resolubilization, and can be compromised by detergents.

## Protocol 4a: denaturing direct-infusion MS

Denaturing direct-infusion ESI mass spectra can usually be obtained by introducing samples to the MS in a mixture of 49.95% HPLC-grade acetonitrile, 49.95% HPLC-grade water, and 0.1% formic acid (v/v). A 60:35:5 ratio of HPLC-grade methanol:water:acetic acid may be used as an alternative and, in some cases, can improve S/N^9,50^. As described above, the use of these organic solvents and acids results in efficient ionization from a droplet’s surface, often allowing MS analysis to be performed using instrumentation parameters typically used for peptides. A more detailed description of instrument parameters for the Bruker SolariX FT-ICR MS used during denaturing direct infusion studies is found in Supplementary Protocol 4a.

## Protocol 4b: native direct-infusion MS

Although native MS protocols may not necessarily produce folded ions that match exactly to their in-solution structures, they can be used to achieve accurate mass measurements of native structures and complexes^51^. Consequently, native direct-infusion MS can provide unique structural information, including the characterization of labile PTMs, metal-binding sites, noncovalent interactions with small molecules, and protein tertiary and quaternary structure. Detergent-free samples can be infused directly in aqueous 2.5 mM ammonium acetate^52^, the same solution used in the final stage of Protocol 2a (concentrations of ammonium acetate up to 500 mM can even be used).

**Fig. 4:**
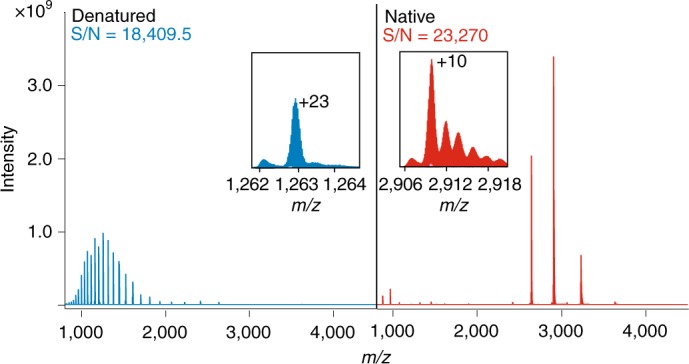
Denatured versus native ESI-MS of carbonic anhydrase. Intensity is scaled to demonstrate the difference between denaturing MS (left) and native MS (right). These spectra were collected on the same instrument using the same concentration (10 µM). Native MS results in lower and fewer charge states, and thus the signals have higher intensity and appear at a higher *m/z*. The inset includes the most abundant charge state and the S/N.

Figure 4 compares mass spectra of carbonic anhydrase in denatured and native states, with the intensity of the base peak in the native sample being about twofold higher than that of the denatured sample. This comparison was repeated in four additional labs on six different instruments to illustrate the possible range of relative intensities (Supplementary Fig. 6, Supplementary Protocol 4b). Membrane protein complexes with MS-compatible detergents can be infused directly from the final solution described in Protocol 2b^45^. To observe native membrane proteins, detergent ions must be removed from the protein–micelle complex by increased collisional activation. This may be achieved through an increase in collision voltage applied to the source or the collision cell (typically 50−200 V), but it could require additional critical parameters that are described in detail by Robinson and coworkers, and in part in Supplementary Protocol 4b^45,53,54^.

## Protocol 5: intact protein analysis using LC-MS

Ionization suppression by excipients and by other proteins generally makes the analysis of multiple proteins and proteoforms by direct infusion intractable. For example, many ‘high-purity’ proteins (as judged by SDS-PAGE) contain numerous proteoforms that cannot be reliably detected and quantitatively assessed without up-front separation^55,56^. Liquid phase separation approaches, including LC (for example, reversed-phase (RP), size-exclusion, ion exchange, chromatofocusing) and capillary electrophoresis techniques (for example, capillary zone electrophoresis, capillary isoelectric focusing) can remove excipients and provide the resolving power for deep characterization of proteins. As directed in the decision tree (Fig. 2), separation of particularly complex samples (>100 proteins) requires an additional dimension of separation before LC-MS. Supplementary Fig. 2 shows the use of GELFrEE separation prior to LC, which fractionates samples on the basis of protein molecular weights and has resulted in the largest number of characterized proteoforms to date^57^.

### Protocol 5a: LC-MS of soluble proteins

RP-LC is recommended for all samples containing more than five unique proteins but is also a viable option for samples with fewer proteins, provided they do not contain high salt concentrations (>1 M) or harsh detergents. The recommended reversed-phase LC protocol is described in Supplementary Protocol 5a and at http://nrtdp.northwestern.edu/protocols/.

**Fig. 5:**
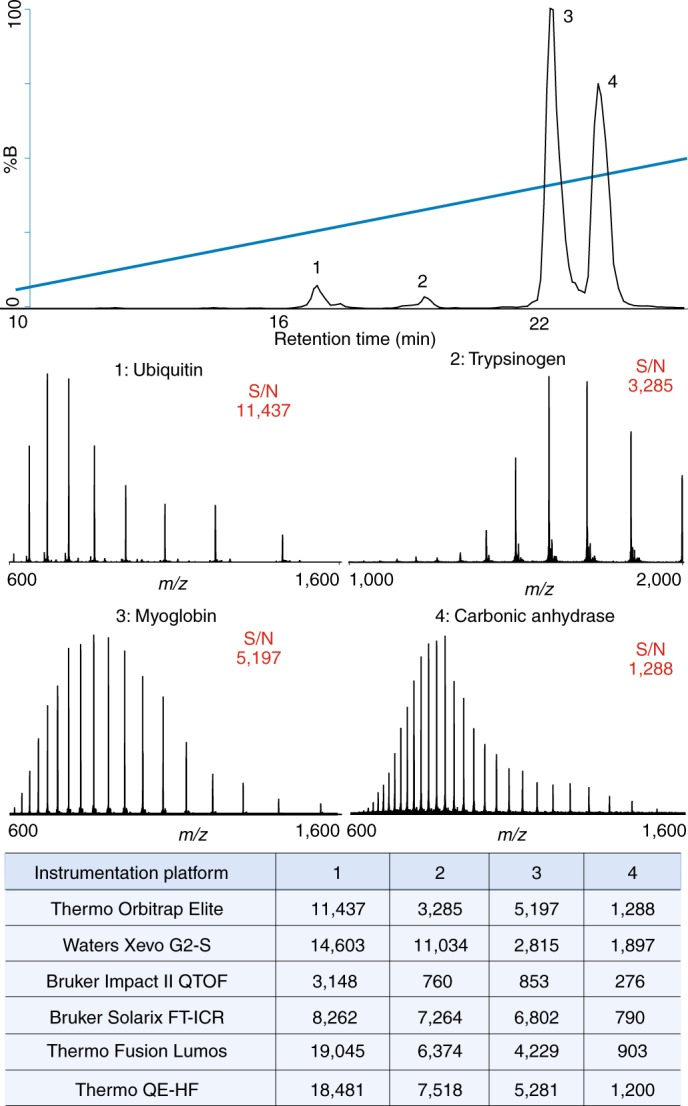
LC-MS of protein standard mixture prepared following Protocol 5a and separated on a Dionex UPLC with a Thermo Orbitrap Elite system using PLRP-S stationary phase. Final concentrations of each protein loaded onto the column were 0.14 pmol ubiquitin, 0.49 pmol trypsinogen, 1.09 pmol myoglobin and 0.64 pmol carbonic anhydrase (top). Summary of S/N values calculated for each protein on all instrumentation platforms using the given SOP (bottom) including Dionex Ultimate 3000–Thermo Orbitrap Elite, Waters Acquity–Xevo G2-S QTOF, Waters nanoAcquity–Bruker Impact II QTOF, Waters nanoAcquity–Bruker SolariX FT-ICR, Dionex Ultimate 3000–Thermo Fusion Lumos, and Dionex Ultimate 3000–Thermo QE-HF. As described, S/N calculations differ per manufacturer and do not reflect absolute performance.

Figure 5 demonstrates that sufficient intact MS signal was attained, and four unique chromatographic peaks were observed, using Protocol 5a with a PLRP-S stationary phase (1,000-Å pore size, 5-µm particle size) on a Dionex UPLC coupled to a Thermo Orbitrap Elite. We provide benchmarks for this standard operating procedure (SOP), as well as for additional data acquired using Monolithic and C4 stationary phases, for six widely used platforms (Waters Xevo G2-S QTOF, Supplementary Fig. 7; Bruker Impact II QTOF and Bruker SolariX FT-ICR, Supplementary Fig. 8; Thermo Orbitrap Elite, Thermo Orbitrap Fusion Lumos, and Thermo Orbitrap QE-HF, Supplementary Figs. 9 and 10). To allow users to compare their performance with that of experienced operators using instruments that are operating within specifications, we report S/N for the platforms used here (Fig. 5). However, instrument vendors use proprietary, non-standardized techniques to preprocess data, display data and determine S/N, and, as a result, our data cannot be used for a cross-platform comparison. As an example of a viable alternative method that is notably better suited for proteoforms with similar mass and RP-LC retention (for example, deamidation), we provide a separation of the same protein mix using capillary zone electrophoresis (Supplementary Fig. 11).

### Protocol 5b: intact membrane protein LC-MS

Denaturing LC-MS of intact membrane proteins is not straightforward because of their inherent hydrophobicity^58,59^. Whitelegge et al. provided the earliest example of denaturing LC-MS of membrane proteins using high concentrations of mobile phase additives and demonstrated that ESI of membrane proteins could achieve the 0.01% mass accuracy benchmark established for ESI of soluble proteins^58^. For thorough reviews of the current state of membrane protein analysis via LC-MS^60,61^ and the corresponding protocols, we direct readers to refs. ^60–62^.

**Fig. 6:**
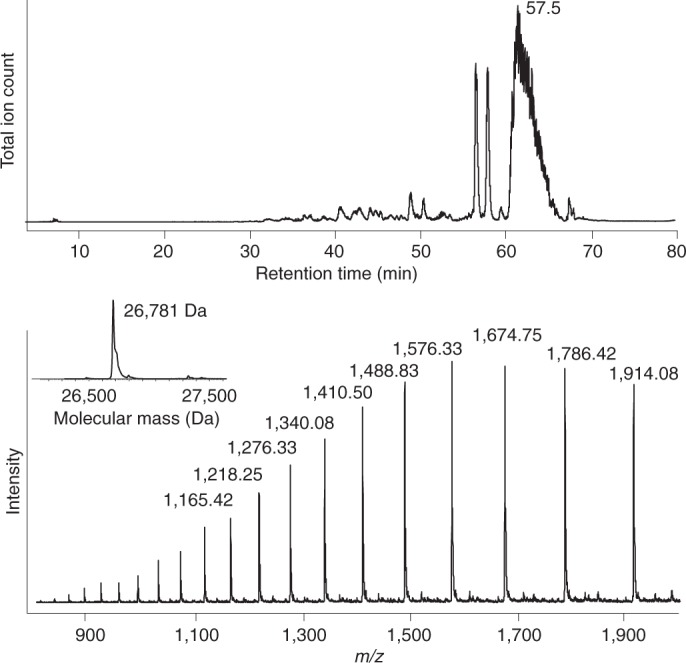
LC-MS of bacteriorhodopsin-containing purple membrane of *H*. *salinarum* prepared following Protocol 5b and analyzed on an Agilent HPLC system coupled to a Thermo linear ion trap (LTQ) mass spectrometer. Proteins were separated using an Agilent PLRP-S 300 Å, 2.1 × 150 mm, 3 µm. Supplementary Fig. 12 demonstrates this analysis on four additional instrumentation platforms.

Current denaturing LC-MS methods for membrane proteins use either size-exclusion^63,64^ or reversed-phase separation. Owing to the ease of implementation across a variety of MS platforms, we suggest analysis via reversed-phase LC-MS using a polystyrene-divinyl benzene co-polymer stationary phase (PLRP-S, 300 Å, Agilent). We do not recommend the use of long chain bonded stationary phases such as C8 and C18, as membrane proteins are likely to be retained on the column. As an example, we solubilized enriched bacteriorhodopsin from *H. salinarum* (Sigma B0184) in 88% formic acid to separate the protein from lipid contaminants. To avoid the risk of formic acid adduction (+28 Da), samples are immediately injected onto the column and solvent exchanged to much lower acid concentrations (0.1%). In the case of membrane protein preparations containing high enough concentrations of lipid contaminants to confound analysis or damage the column, we recommend precipitation following Protocol 3 before analysis. Proteins are eluted using an increasing gradient of 49.95% acetonitrile, 49.95% isopropanol, 0.1% formic acid. Figure 6 shows the analysis of denatured bacteriorhodopsin of *H. salinarum* following this protocol. Although elution efficiency for some integral proteins may fall well below 100%, PLRP-S columns can be regenerated with 90% formic acid injections. This protocol was performed in five labs on five different instrument platforms (Supplementary Fig. 12, Supplementary Protocol 5b). An example of an alternative LC-MS method using a more common stationary phase (ZORBAX RRHD 300SB-C3) is provided for aquaporin Z in Supplementary Fig. 5b.

## Special methodological considerations for intact antibody mass spectrometry

With the increasing development of biotherapeutics and biosimilars in the pharmaceutical industry, and an increasingly stringent route to regulatory approvals, there is a growing need for intact antibody MS. Every protocol presented here can be applied to the analysis of intact antibodies (Fig. 3, Supplementary Fig. 13, Supplementary Note 4). However, as antibodies are relatively large and signal spreading increases in proportion to protein size, we recommend against the use of Protocol 1 (dilution) for any regulatory filing.

## Discussion

The IPMS decision tree (Fig. 2) guides practitioners of all levels toward broadly applicable methods to obtain high-quality intact mass spectra from any protein sample. The protocols described here have been scrutinized and optimized in over ten expert intact protein MS labs, and successfully applied in laboratories without experience in intact protein MS. We hope that these protocols will enable any research group to adopt intact protein mass analysis.

The accurate mass measurement of an intact protein is the *sine qua non* of top-down mass spectrometry, which can characterize how proteoforms interact and identify PTMs that are lost in other analyses. High-throughput top-down analysis of whole proteomes has proven successful in the unambiguous identification of hundreds of proteins and proteoforms from a single biological sample^65^ and revealed prevalent yet previously uncharacterized biologically relevant modifications^66^. Quantitative top-down proteomics has been used to identify disease-relevant differences in protein levels, an encouraging step forward in the field of proteomics-based personalized medicine^67^. Additionally, by using native mass spectrometry following the top-down workflow, one can observe previously unknown protein–protein interactions, protein–ligand binding, protein–cofactor association and protein-complex stoichiometry, and assess their relationships to important biological pathways^68^. We believe that by starting with intact mass analysis, using these intact protein MS protocols coupled to top-down MS analysis, and by identifying proteoforms rather than proteins, scientists can gain new insights into the human proteome. We also hope that these protocols serve as a starting point for users to push, even further, the current limits of high-molecular-weight mass spectrometry.

All general protocols are available as Supplementary Protocols.

## Supplementary Information

### Integrated supplementary information

Supplementary Figure 1 Signal suppression curves of common components. These components are outlined in Fig. 1c. The x-axis represents an increasing concentration of interfering substance [C] and the y-axis represents the fraction of signal lost. Each spectrum was collected in triplicate. S/N was calculated as described in the Online Methods. Standard deviation of each data point was calculated and used to produce error bars. The least-squares fitting (LSF) calculation is included to show the quality of fit to the equation.

Supplementary Figure 2 Fractionation of human whole-cell lysate prior to top-down mass spectrometry. Human colorectal cancer cells were lysed and constituent proteins quantified by the methods described by Anderson *et. al*.^1^ Aliquots of lysate containing 400 µg were precipitated in acetone, resuspended in 1% SDS containing 50 mM DTT, and resolved on 8% T (a.) or 10% T (b.) gel-eluted liquid fraction entrapment electrophoresis (GELFrEE) cartridges following the respective manufacturer’s protocols (GELFrEE 8100 Fractionation System, Expedeon, Inc.). Upon collection of MW-based fractions, 10 µL aliquots were resolved by SDS-PAGE and visualized by AgNO_3_ stain^2^ to gauge protein content and quality of resolution. (a,b) Note that the MW ranges of f1 (purple box) and subsequent fractions differ depending on the GELFrEE cartridge selected. While 8% cartridges (a.) are recommended for quantitative high-throughput top-down MS applications or analysis of higher-MW proteins, 10% (b.) cartridges provide superior resolution in the 5-30 kDa MW range for qualitative high-throughput applications. 1. Anderson, L.C. et al. Identification and Characterization of Human Proteoforms by Top-Down LC-21 Tesla FT-ICR Mass Spectrometry. J Proteome Res 16, 1087-1096 (2017). 2. Shevchenko, A., Wilm, M., Vorm, O. & Mann, M. Mass spectrometric sequencing of proteins silver-stained polyacrylamide gels. Anal Chem 68, 850-858 (1996).

Supplementary Figure 3 Antibody Buffer and Gentle Elution Buffer Ablate MS Signal; MWCO-Ultrafiltration and Precipitation Rescue Signal. Buffers included (a.) Thermo Gentle Elution Buffer (containing molar salt concentration), and (b.) Antibody buffer (10 mM Arginine, 10 mM Tris HCl, 10 mM histidine, 10 mM potassium phosphate, 10 mM citric acid, pH 5.5). All the above spectra were obtained using a Bruker SolariX FT-ICR mass spectrometer, 9.4T.

Supplementary Figure 4 Sample Preparation of Protein Mixture following Protocol 3 (MWCO-Ultrafiltration). These samples were analyzed by direct infusion on a (a.) Waters Xevo G2-S QTOF and a (b.) Thermo Q Exactive Plus.

Supplementary Figure 5 Native vs. Denatured MS of AquaporinZ (AqpZ) from E. coli. Native spectrum was acquired on a Waters Synapt G1 Q-TOF with nanoESI via direct infusion while denatured spectra was acquired on a Waters Synapt G1 Q-TOF via nanoESI-LC-MS. The native sample (a.) and the denatured sample (b.) was acquired at a concentration of 10 µM. *Denotes the most abundant charge state. 24268.7 Da is the deconvoluted mass of the unmodified AqpZ monomer. Formylated AqpZ was also detected with a mass of 24,296.4 Da. ^‡^Five native tetramer masses were observed corresponding to five unique combinations of formylated and unformylated monomers.^85^

Supplementary Figure 6 Denaturing vs. Native Analysis of Carbonic Anhydrase. Both denaturing and native analysis of carbonic anhydrase was run on a (a.) Thermo Q Exactive HF MS (b.) Bruker 15T SolariX FT-ICR MS, (c.) Bruker 12T SolariX FT-ICR MS, (d.) Bruker maXis II ETD Q-TOF, (e.) Bruker 15T SolariX FT-ICR MS, (f.) Waters Synapt G2Si MS.

Supplementary Figure 7 LC-MS of protein standard mixture run on Waters Acquity-Xevo G2-S QTOF. Samples were prepared following the given SOP and separated using a PLRP-S (top panel of a, b, and c) or a C4 (bottom panel of a, b, and c) stationary phase. (a.) The final concentrations of each protein loaded onto the column were; 14 pmol ubiquitin, 49 pmol trypsinogen, 109 pmol myoglobin, and 64 pmol carbonic anhydrase. (b.) The final concentrations of each protein loaded onto the column were; 1.4 pmol ubiquitin, 4.9 pmol trypsinogen, 10.9 pmol myoglobin, and 6.4 pmol carbonic anhydrase. (c.) The final concentrations of each protein loaded onto the column were; 0.14 pmol ubiquitin, 0.49 pmol trypsinogen, 1.09 pmol myoglobin, and 0.64 pmol carbonic anhydrase.

Supplementary Figure 8 LC-MS of protein standard mixture run on Waters nanoAcquity coupled to a Bruker QTOF and a Bruker FT-ICR MS. Samples were prepared following the given SOP and separated using PLRP-S on a Waters nanoAcquity coupled to (a.) a Bruker impact II QTOF and (b.) a Bruker SolariX FT-ICR MS.

Supplementary Figure 9 LC-MS of protein standard mixture run on a Dionex UPLC coupled to three different orbitrap mass spectrometers. Samples were prepared following the given SOP and separated on a Dionex UPLC coupled to (a.) a Thermo Orbitrap Elite (monolithic stationary phase, (b.) a Thermo Orbitrap Fusion Lumos (PLRP-S stationary phase), (c.) a Thermo Orbitrap QE-HF (PLRP-S stationary phase).

Supplementary Figure 10 LC-MS of protein standard mixture run on a Dionex UPLC coupled to a Thermo Orbitrap Fusion Lumos. Samples were prepared following the given SOP, separated on a Dionex UltiMate 3000 RSLCNano System using PLRP-S stationary phase, and analyzed on a Thermo Fusion Lumos. The final concentrations of each protein loaded onto the column were; 0.14 pmol ubiquitin, 0.49 pmol trypsinogen, 1.09 pmol myoglobin, and 0.64 pmol carbonic anhydrase.

Supplementary Figure 11 Capillary Zone Electrophoresis Separation of Protein Mixture. Separated using a prototype CESI-8000 Plus (AB SCIEX) used with a Neutral OptiMS cartridge.

Supplementary Figure 12 LC MS of *Halobacterium salinarum* prepared following Supplemental Protocol 5b. Proteins were separated using a PLRP-S stationary phase (300 Å pore size, 3 µm bead size) and analyzed on a (a.) Waters nanoAcquity interfaced with a Bruker SolariX FT-ICR MS (b.) Waters H-Class Acquity UPLC interfaced with a Waters Xevo G2-S QTOF (c.) Thermo Scientific Vanquish interfaced with a Thermo Orbitrap Q Exactive (d.) Agilent 1290 interfaced with a Thermo Orbitrap Exactive Plus.

Supplementary Figure 13 LC MS of NIST Antibody on a Waters UPLC-QTOF system using C4 stationary phase. These results demonstrate that antibody sample clean-up for intact MS analysis can be achieved without any additional steps required.

### Supplementary information

Supplementary Information Supplementary Figs. 1–13, Supplementary Notes 1–5, and Supplementary Protocols 1–5
