# Supplementary figures and images for: Best practices and benchmarks for intact protein analysis for top-down mass spectrometry
Source: Nat Methods. 2019 Jun 27;16(7):587–94. doi: 10.1038/s41592-019-0457-0 (PMC6719561; doi:10.1038/s41592-019-0457-0)

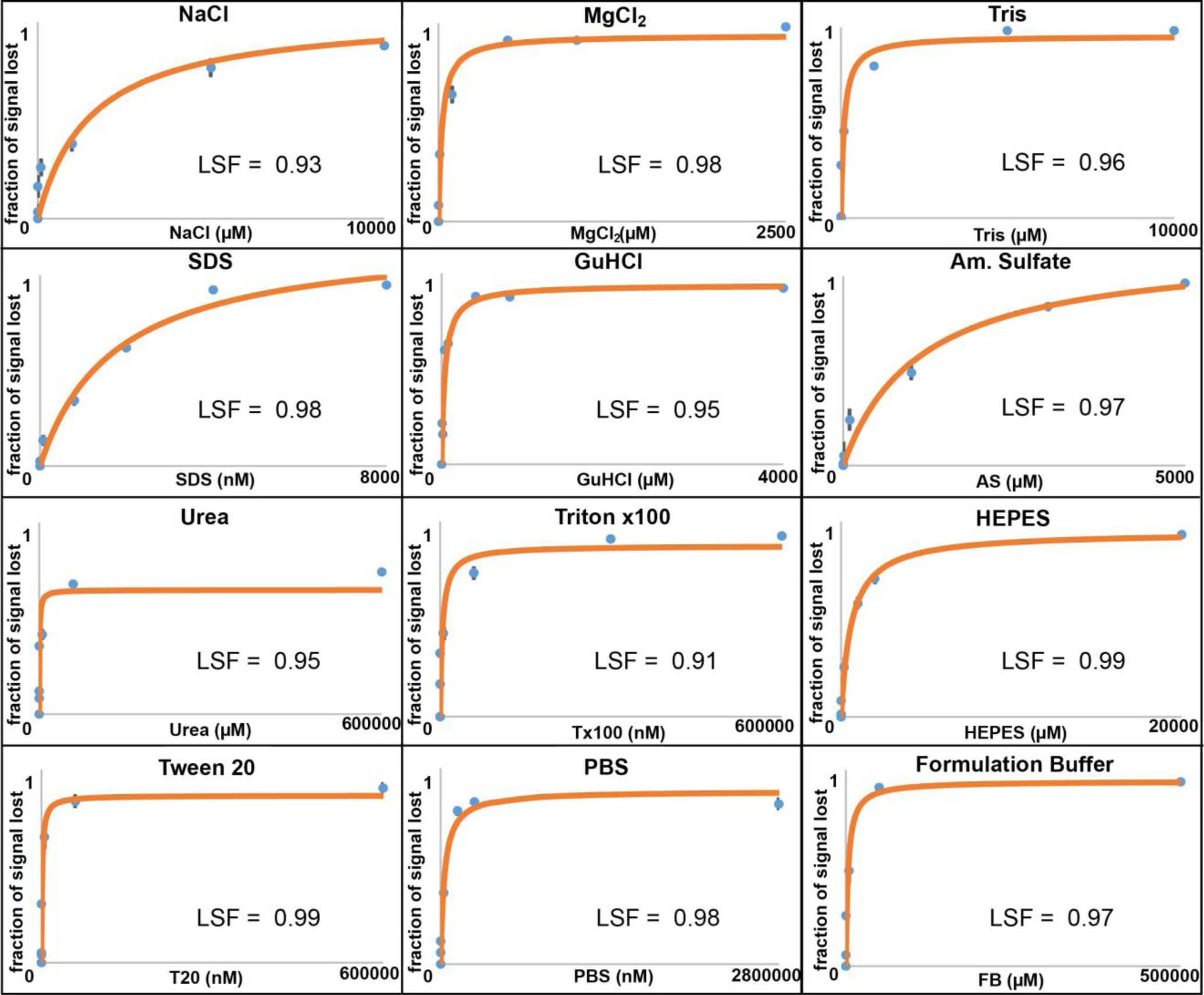

Supplement: Signal suppression curves of common components. — These components are outlined in Fig. 1c. The x-axis represents an increasing concentration of interfering substance [C] and the y-axis represents the fraction of signal lost. Each spectrum was collected in triplicate. S/N was calculated as described in the Online Methods. Standard deviation of each data point was calculated and used to produce error bars. The least-squares fitting (LSF) calculation is included to show the quality of fit to the equation. [file 41592_2019_457_Fig7_ESM.jpg]

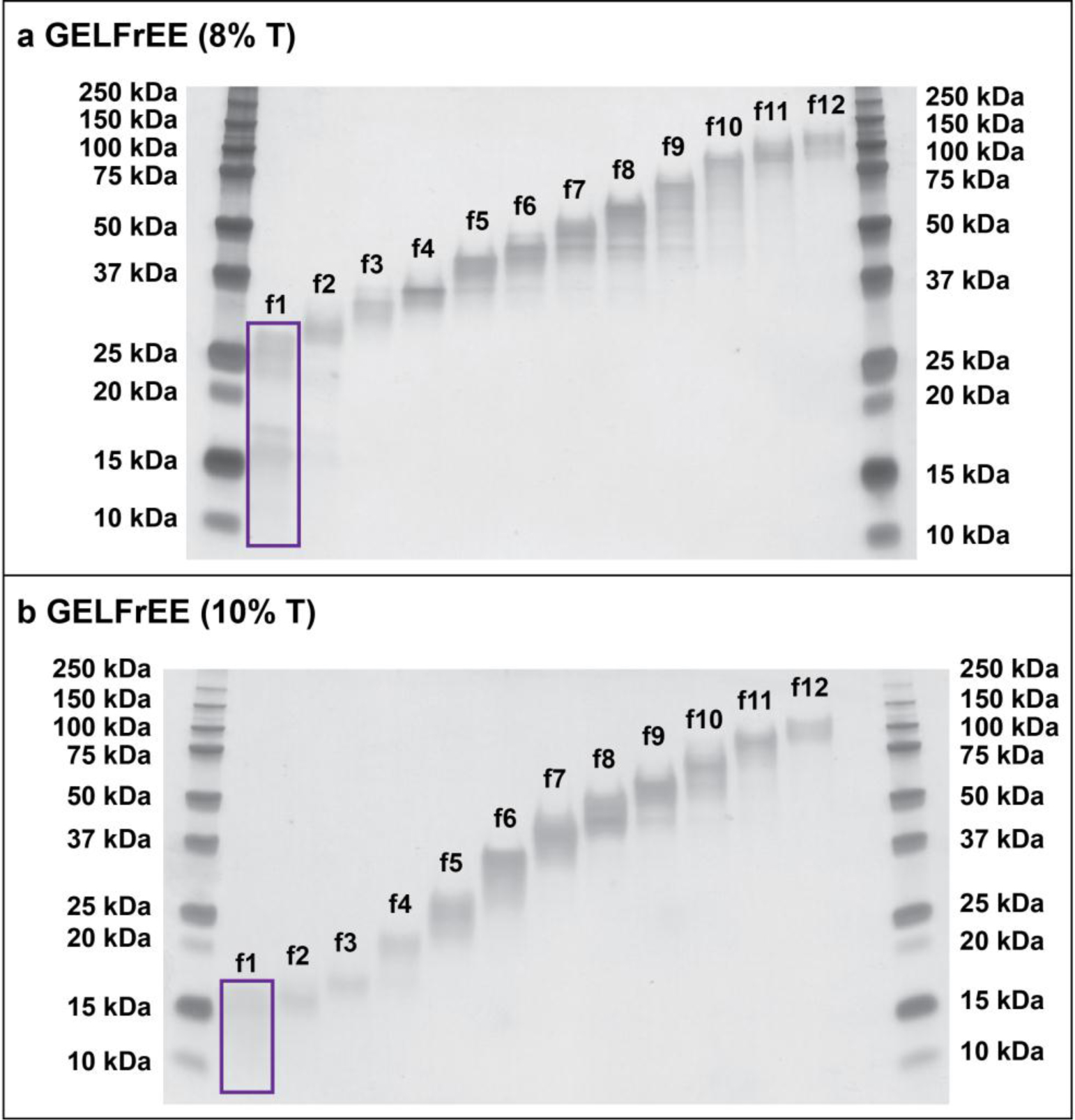

Supplement: Fractionation of human whole-cell lysate prior to top-down mass spectrometry. — Human colorectal cancer cells were lysed and constituent proteins quantified by the methods described by Anderson et. al.1 Aliquots of lysate containing 400 µg were precipitated in acetone, resuspended in 1% SDS containing 50 mM DTT, and resolved on 8% T (a.) or 10% T (b.) gel-eluted liquid fraction entrapment electrophoresis (GELFrEE) cartridges following the respective manufacturer’s protocols (GELFrEE 8100 Fractionation System, Expedeon, Inc.). Upon collection of MW-based fractions, 10 µL aliquots were resolved by SDS-PAGE and visualized by AgNO3 stain2 to gauge protein content and quality of resolution. (a,b) Note that the MW ranges of f1 (purple box) and subsequent fractions differ depending on the GELFrEE cartridge selected. While 8% cartridges (a.) are recommended for quantitative high-throughput top-down MS applications or analysis of higher-MW proteins, 10% (b.) cartridges provide superior resolution in the 5-30 kDa MW range for qualitative high-throughput applications. 1. Anderson, L.C. et al. Identification and Characterization of Human Proteoforms by Top-Down LC-21 Tesla FT-ICR Mass Spectrometry. J Proteome Res 16, 1087-1096 (2017). 2. Shevchenko, A., Wilm, M., Vorm, O. & Mann, M. Mass spectrometric sequencing of proteins silver-stained polyacrylamide gels. Anal Chem 68, 850-858 (1996). [file 41592_2019_457_Fig8_ESM.jpg]

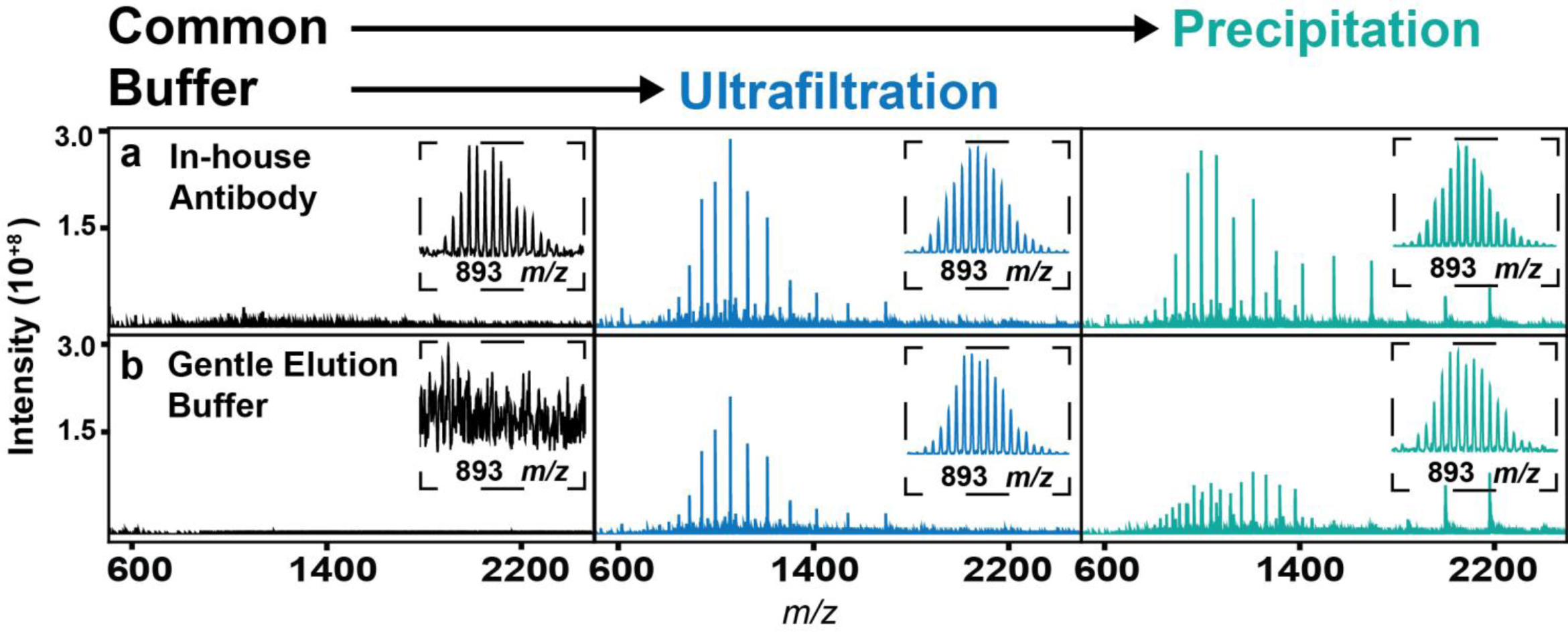

Supplement: Antibody Buffer and Gentle Elution Buffer Ablate MS Signal; MWCO-Ultrafiltration and Precipitation Rescue Signal. — Buffers included (a.) Thermo Gentle Elution Buffer (containing molar salt concentration), and (b.) Antibody buffer (10 mM Arginine, 10 mM Tris HCl, 10 mM histidine, 10 mM potassium phosphate, 10 mM citric acid, pH 5.5). All the above spectra were obtained using a Bruker SolariX FT-ICR mass spectrometer, 9.4T. [file 41592_2019_457_Fig9_ESM.jpg]

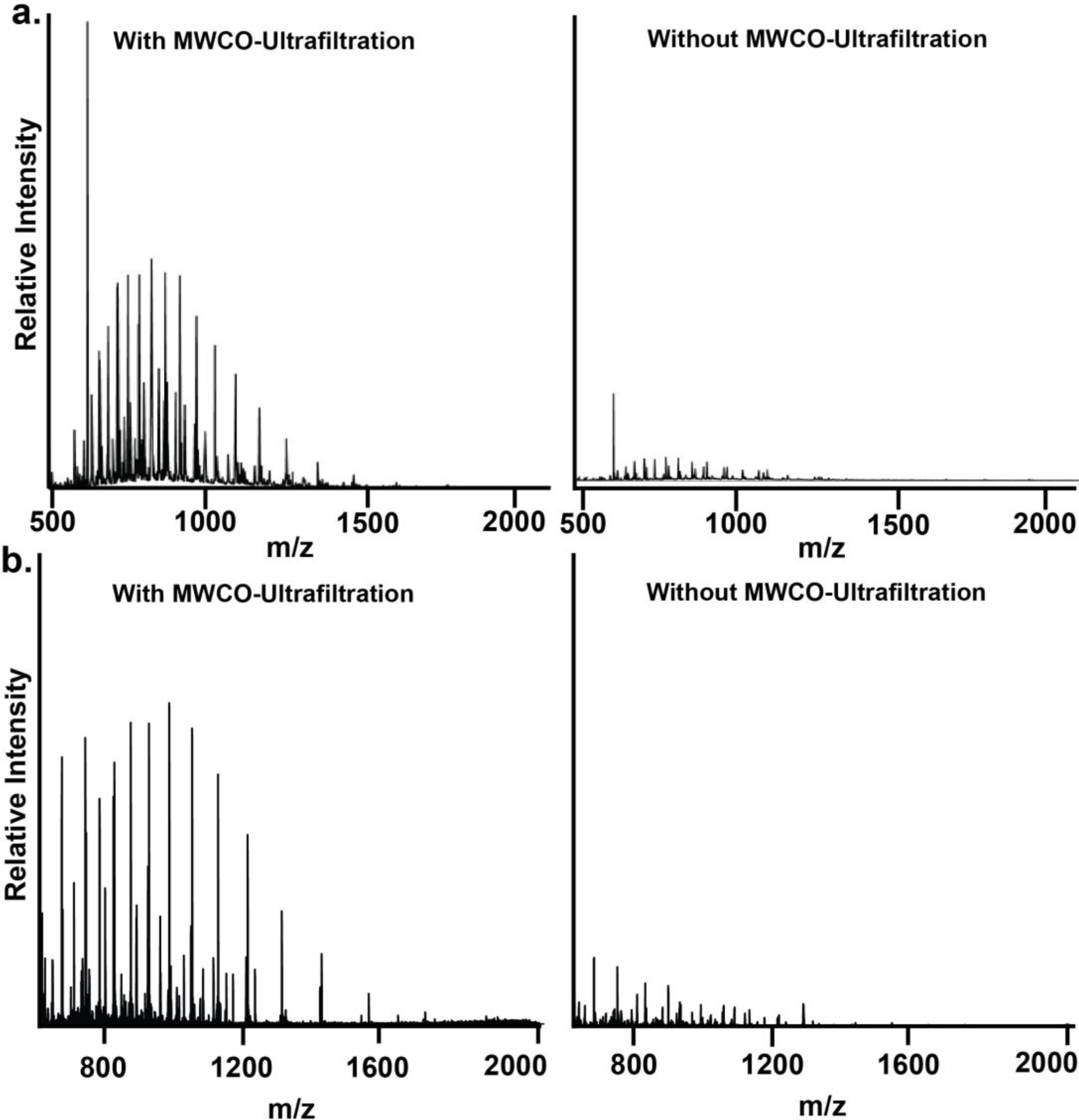

Supplement: Sample Preparation of Protein Mixture following Protocol 3 (MWCO-Ultrafiltration). — These samples were analyzed by direct infusion on a (a.) Waters Xevo G2-S QTOF and a (b.) Thermo Q Exactive Plus. [file 41592_2019_457_Fig10_ESM.jpg]

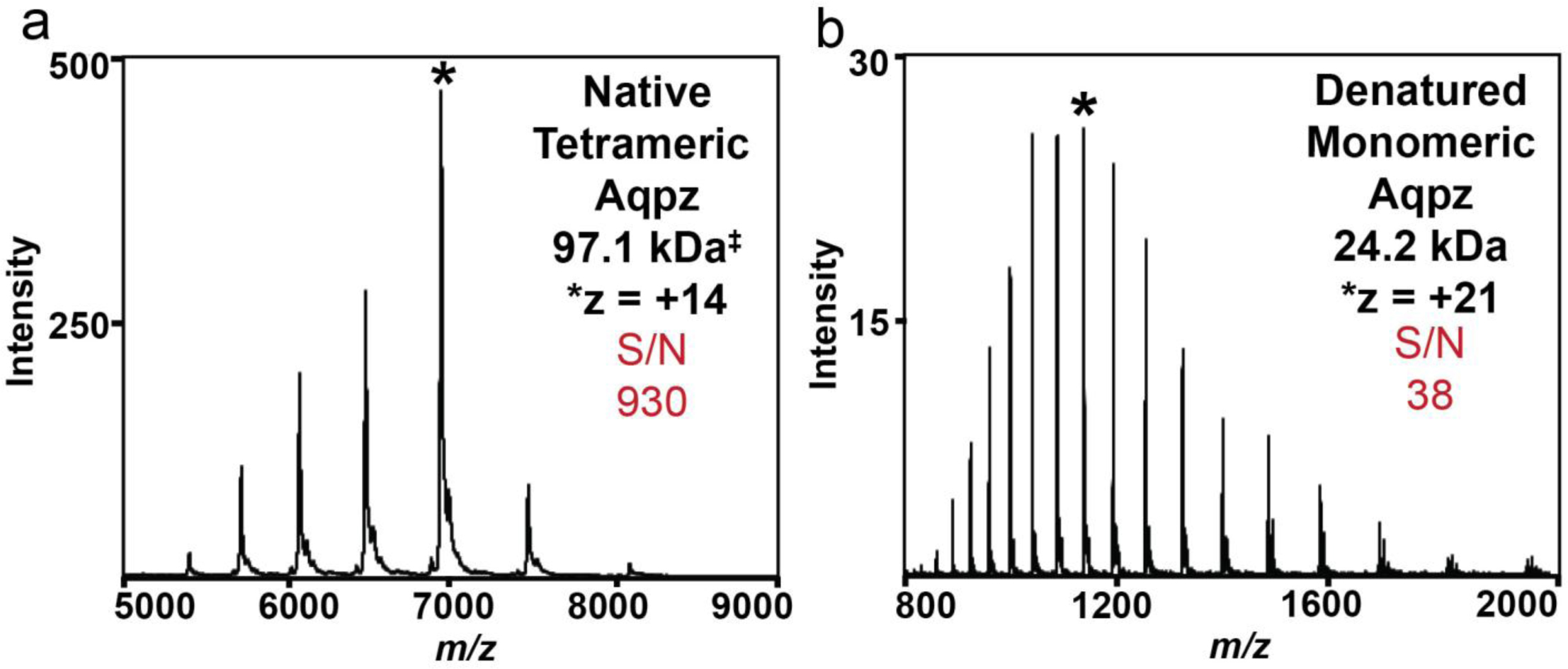

Supplement: Native vs. Denatured MS of AquaporinZ (AqpZ) from E. coli. — Native spectrum was acquired on a Waters Synapt G1 Q-TOF with nanoESI via direct infusion while denatured spectra was acquired on a Waters Synapt G1 Q-TOF via nanoESI-LC-MS. The native sample (a.) and the denatured sample (b.) was acquired at a concentration of 10 µM. *Denotes the most abundant charge state. 24268.7 Da is the deconvoluted mass of the unmodified AqpZ monomer. Formylated AqpZ was also detected with a mass of 24,296.4 Da. ‡Five native tetramer masses were observed corresponding to five unique combinations of formylated and unformylated monomers.85 [file 41592_2019_457_Fig11_ESM.jpg]

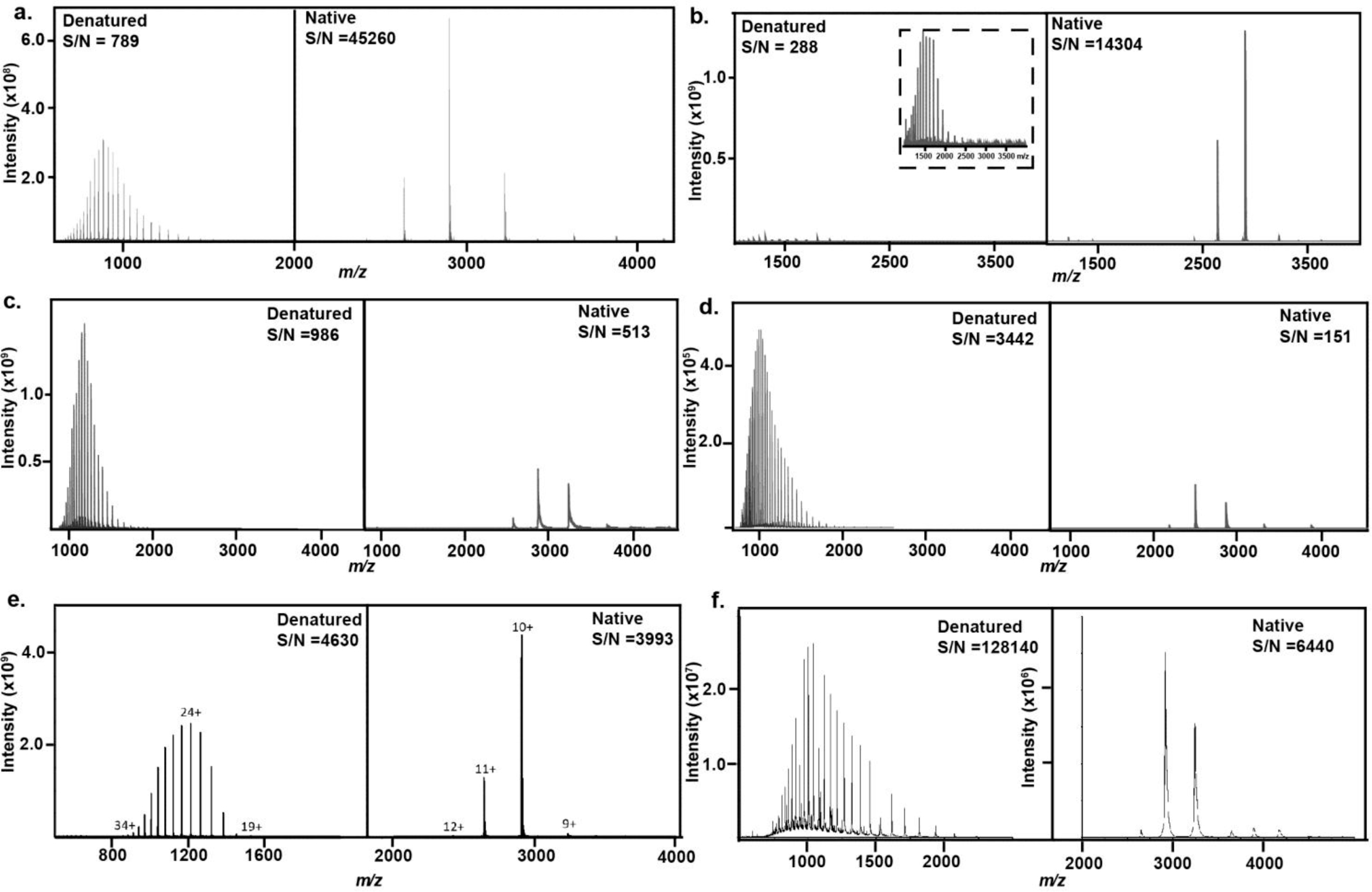

Supplement: Denaturing vs. Native Analysis of Carbonic Anhydrase. — Both denaturing and native analysis of carbonic anhydrase was run on a (a.) Thermo Q Exactive HF MS (b.) Bruker 15T SolariX FT-ICR MS, (c.) Bruker 12T SolariX FT-ICR MS, (d.) Bruker maXis II ETD Q-TOF, (e.) Bruker 15T SolariX FT-ICR MS, (f.) Waters Synapt G2Si MS. [file 41592_2019_457_Fig12_ESM.jpg]

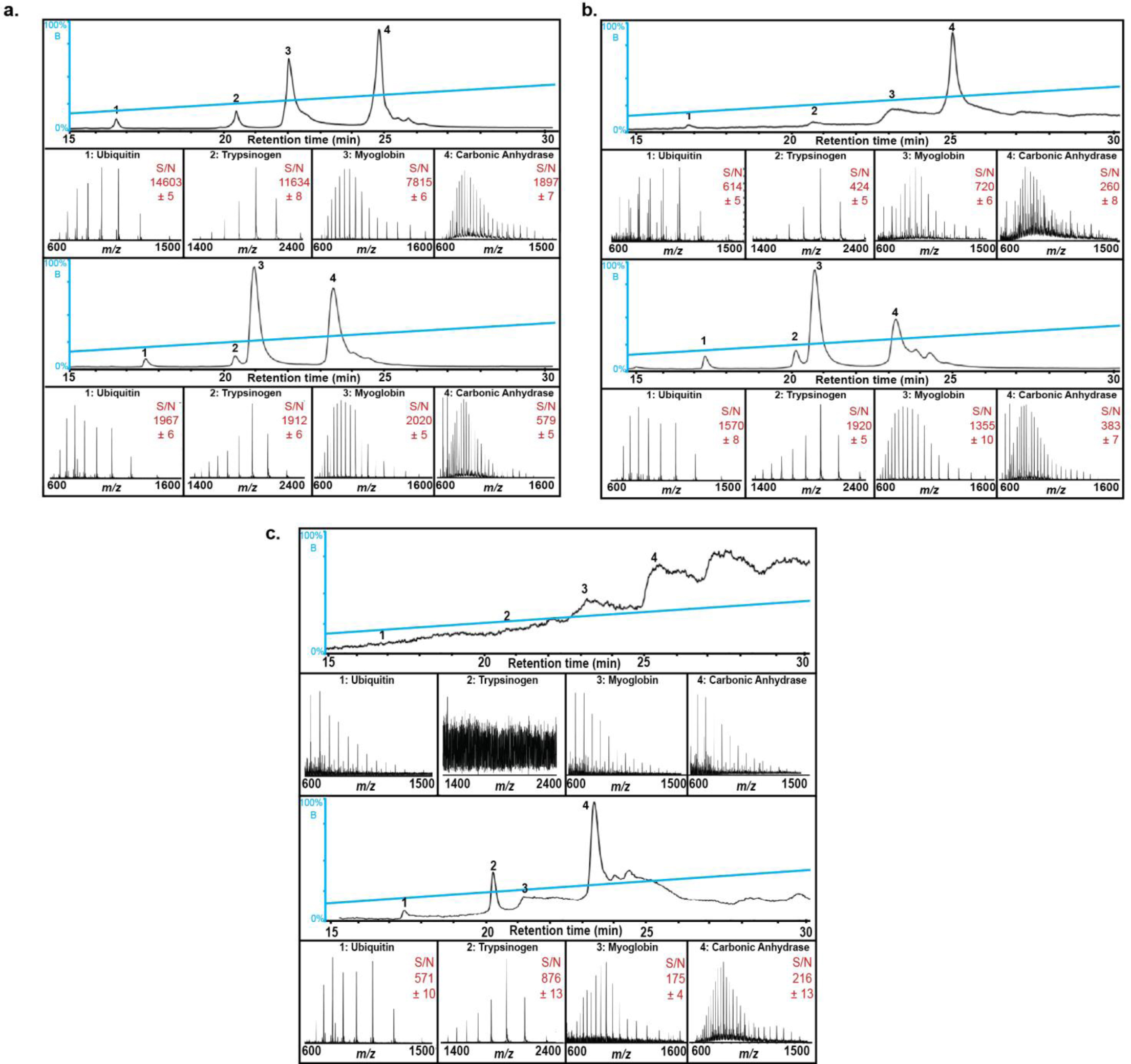

Supplement: LC-MS of protein standard mixture run on Waters Acquity-Xevo G2-S QTOF. — Samples were prepared following the given SOP and separated using a PLRP-S (top panel of a, b, and c) or a C4 (bottom panel of a, b, and c) stationary phase. (a.) The final concentrations of each protein loaded onto the column were; 14 pmol ubiquitin, 49 pmol trypsinogen, 109 pmol myoglobin, and 64 pmol carbonic anhydrase. (b.) The final concentrations of each protein loaded onto the column were; 1.4 pmol ubiquitin, 4.9 pmol trypsinogen, 10.9 pmol myoglobin, and 6.4 pmol carbonic anhydrase. (c.) The final concentrations of each protein loaded onto the column were; 0.14 pmol ubiquitin, 0.49 pmol trypsinogen, 1.09 pmol myoglobin, and 0.64 pmol carbonic anhydrase. [file 41592_2019_457_Fig13_ESM.jpg]

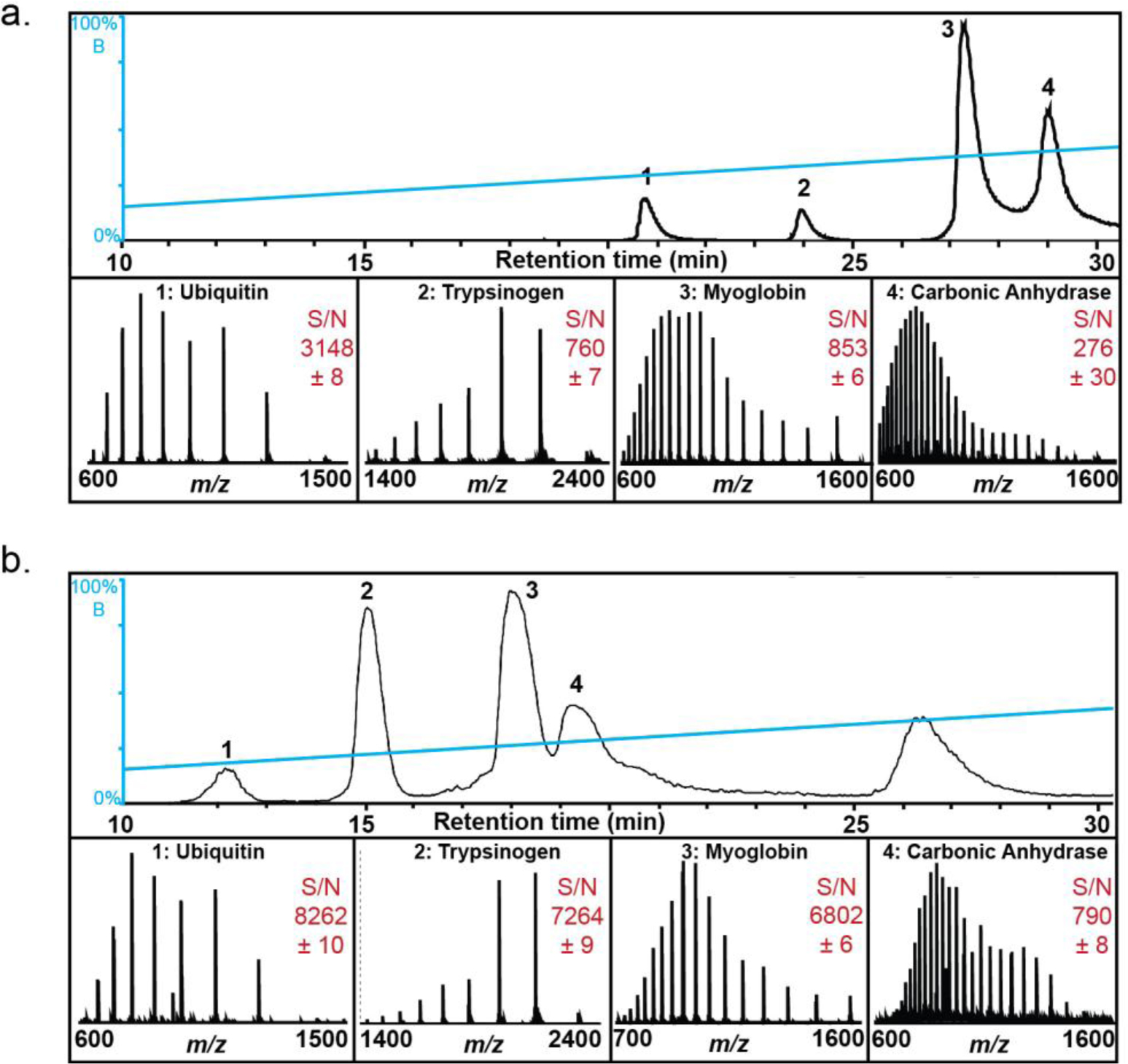

Supplement: LC-MS of protein standard mixture run on Waters nanoAcquity coupled to a Bruker QTOF and a Bruker FT-ICR MS. — Samples were prepared following the given SOP and separated using PLRP-S on a Waters nanoAcquity coupled to (a.) a Bruker impact II QTOF and (b.) a Bruker SolariX FT-ICR MS. [file 41592_2019_457_Fig14_ESM.jpg]

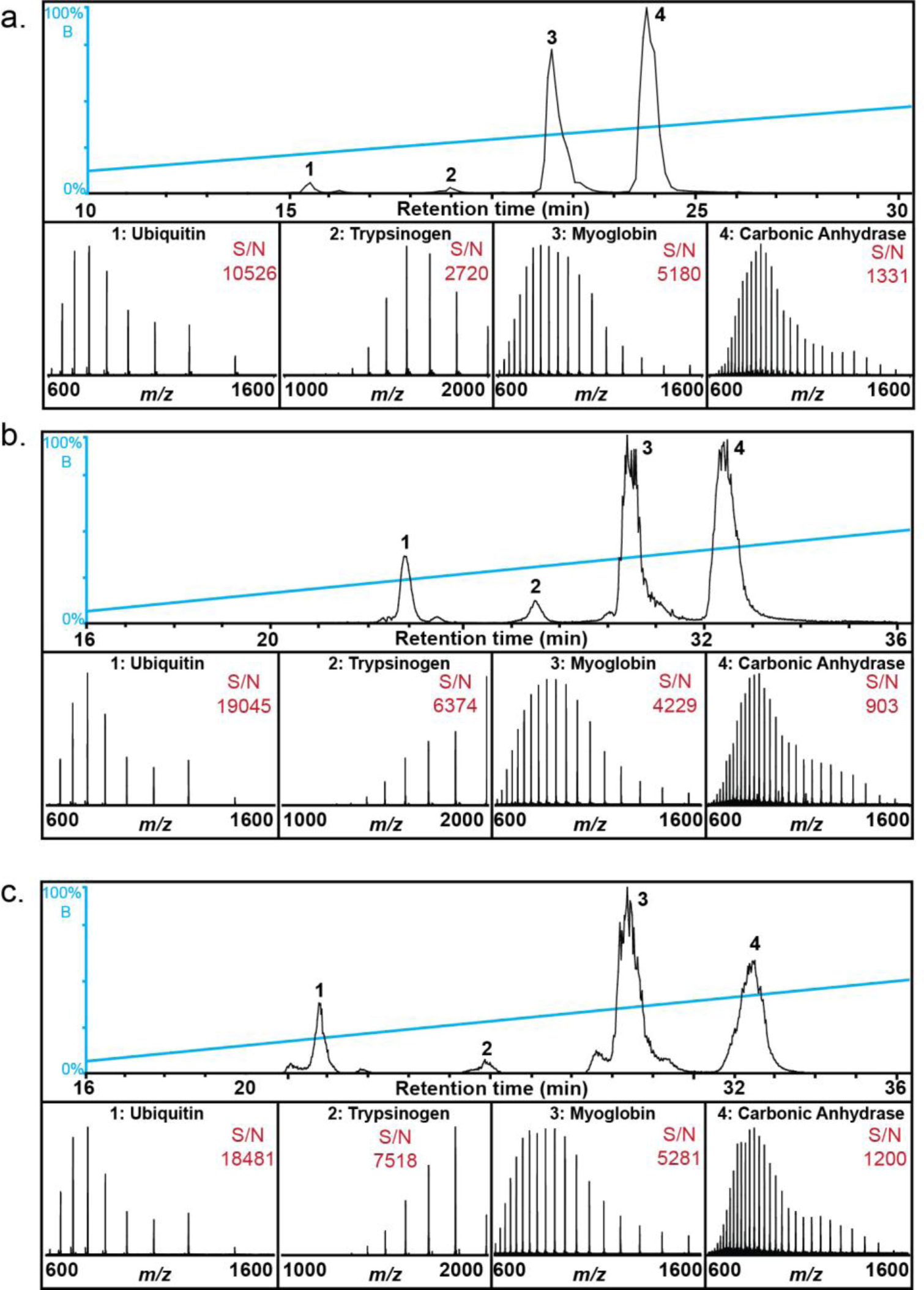

Supplement: LC-MS of protein standard mixture run on a Dionex UPLC coupled to three different orbitrap mass spectrometers. — Samples were prepared following the given SOP and separated on a Dionex UPLC coupled to (a.) a Thermo Orbitrap Elite (monolithic stationary phase, (b.) a Thermo Orbitrap Fusion Lumos (PLRP-S stationary phase), (c.) a Thermo Orbitrap QE-HF (PLRP-S stationary phase). [file 41592_2019_457_Fig15_ESM.jpg]

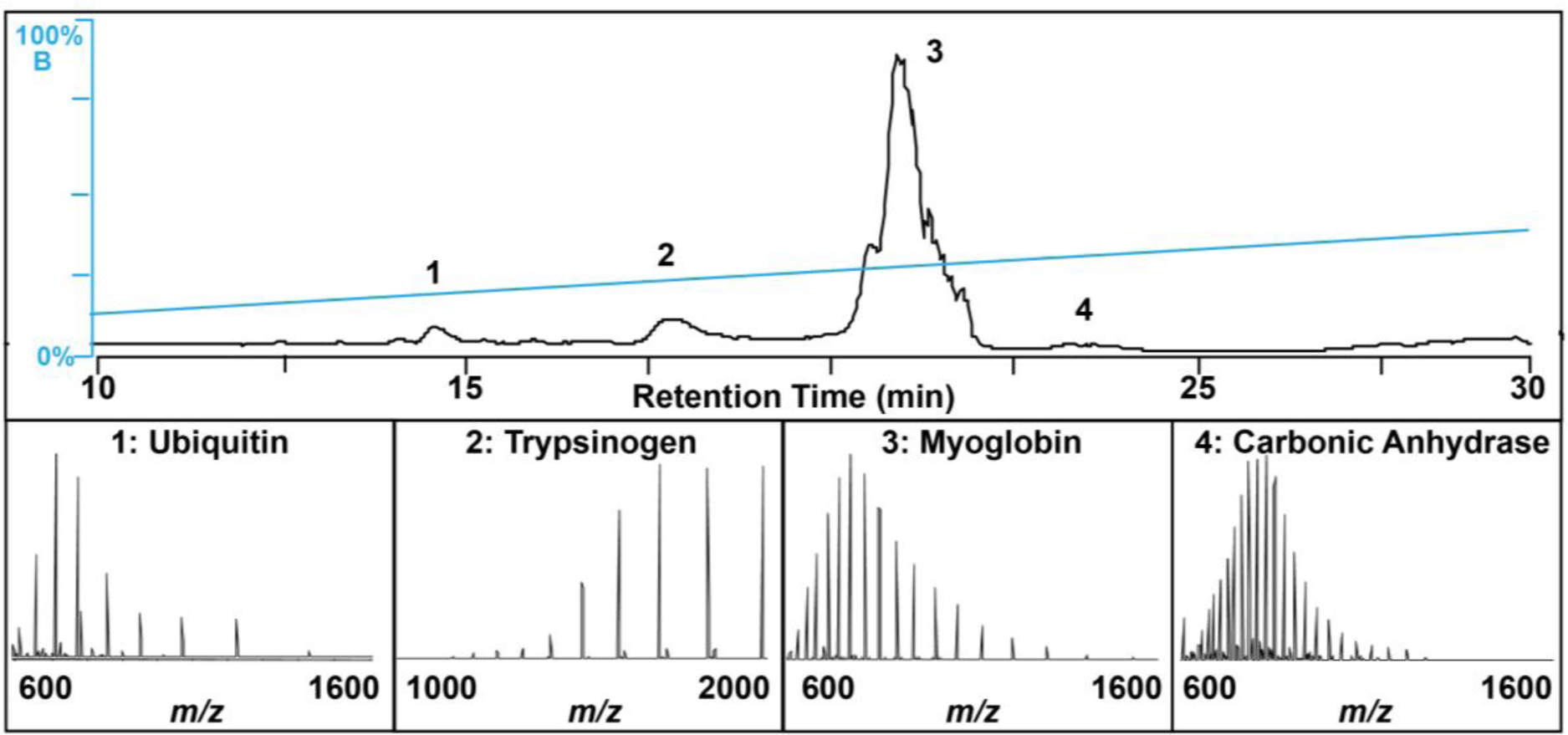

Supplement: LC-MS of protein standard mixture run on a Dionex UPLC coupled to a Thermo Orbitrap Fusion Lumos. — Samples were prepared following the given SOP, separated on a Dionex UltiMate 3000 RSLCNano System using PLRP-S stationary phase, and analyzed on a Thermo Fusion Lumos. The final concentrations of each protein loaded onto the column were; 0.14 pmol ubiquitin, 0.49 pmol trypsinogen, 1.09 pmol myoglobin, and 0.64 pmol carbonic anhydrase. [file 41592_2019_457_Fig16_ESM.jpg]

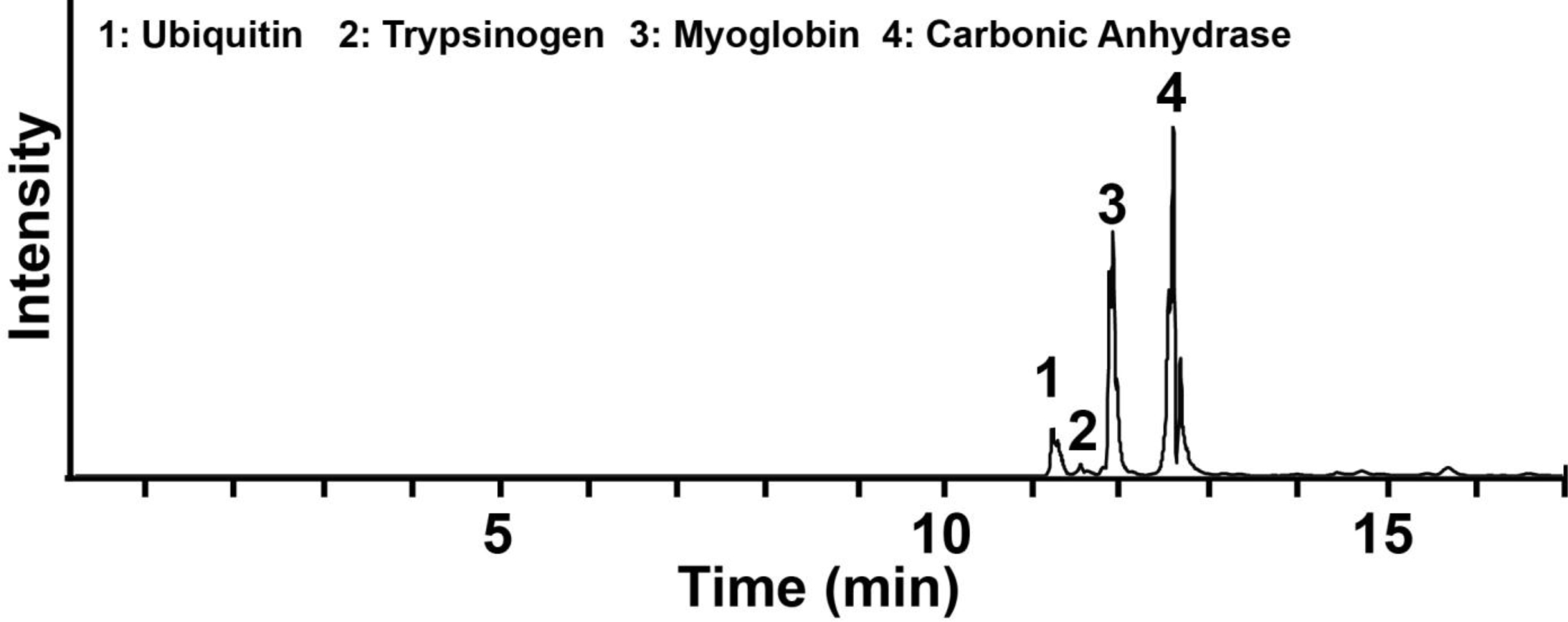

Supplement: Capillary Zone Electrophoresis Separation of Protein Mixture. — Separated using a prototype CESI-8000 Plus (AB SCIEX) used with a Neutral OptiMS cartridge. [file 41592_2019_457_Fig17_ESM.jpg]

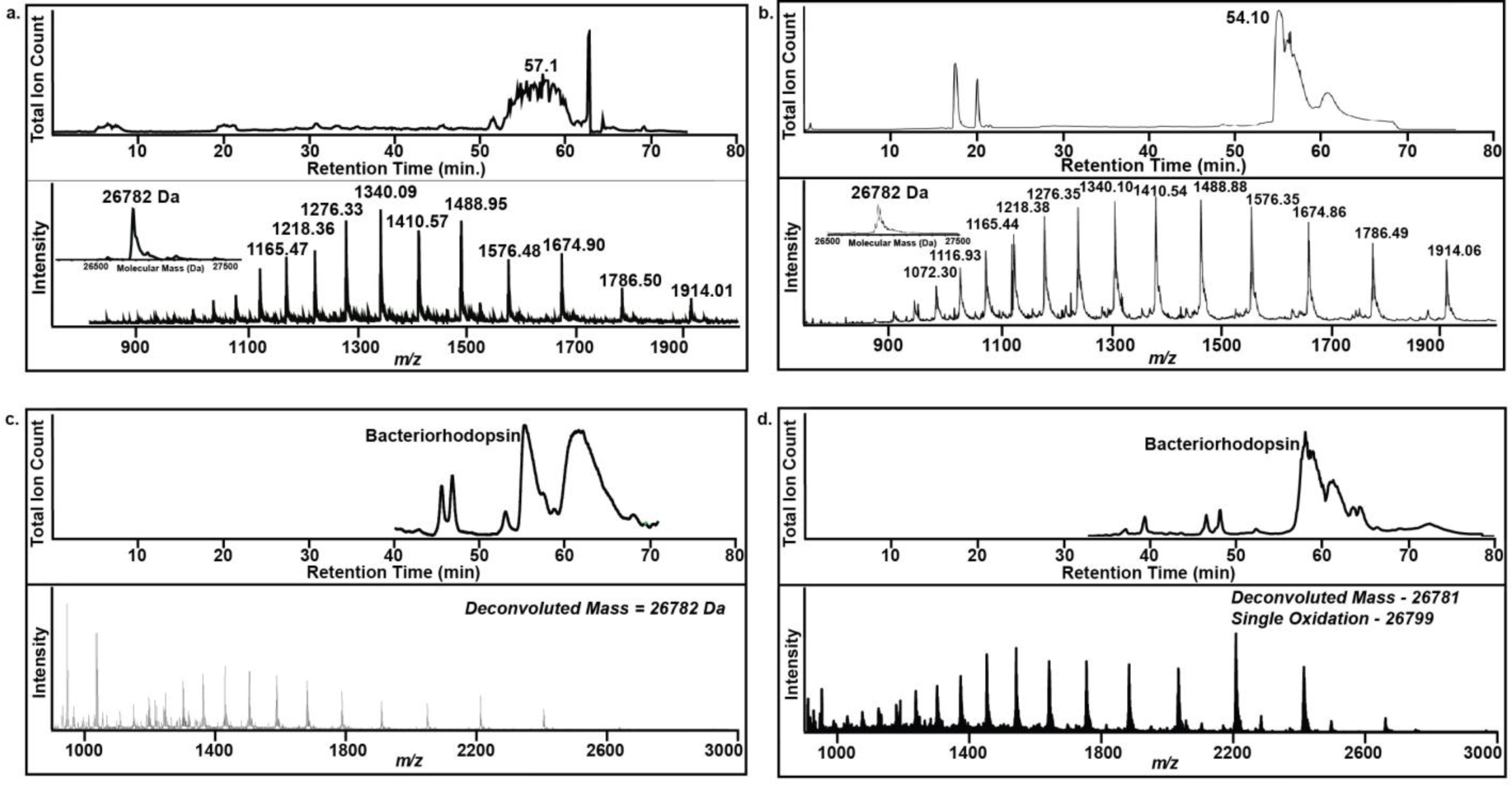

Supplement: LC MS of Halobacterium salinarum prepared following Supplemental Protocol 5b. — Proteins were separated using a PLRP-S stationary phase (300 Å pore size, 3 µm bead size) and analyzed on a (a.) Waters nanoAcquity interfaced with a Bruker SolariX FT-ICR MS (b.) Waters H-Class Acquity UPLC interfaced with a Waters Xevo G2-S QTOF (c.) Thermo Scientific Vanquish interfaced with a Thermo Orbitrap Q Exactive (d.) Agilent 1290 interfaced with a Thermo Orbitrap Exactive Plus. [file 41592_2019_457_Fig18_ESM.jpg]

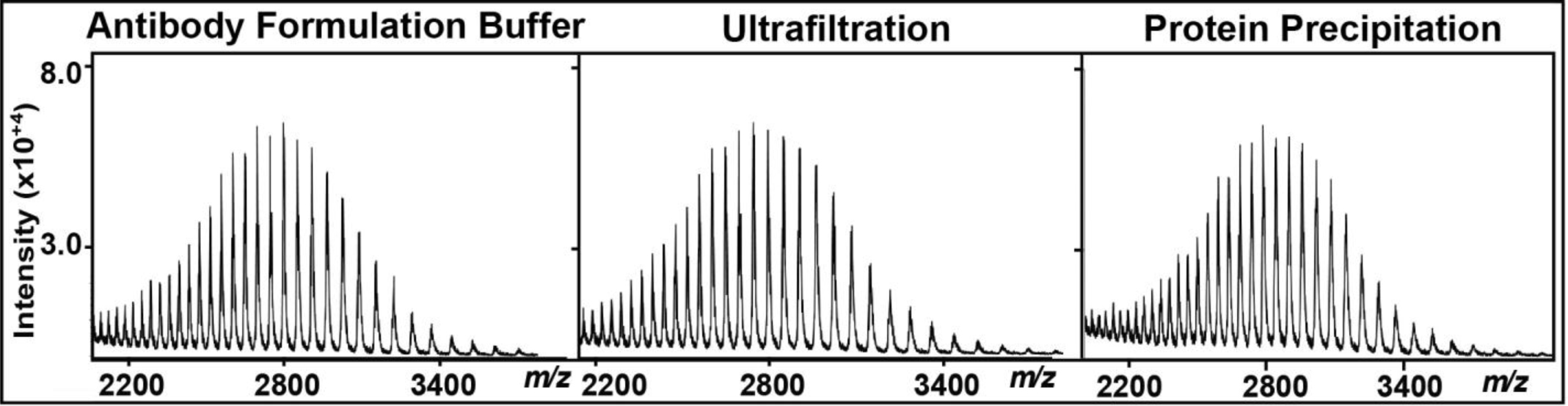

Supplement: LC MS of NIST Antibody on a Waters UPLC-QTOF system using C4 stationary phase. — These results demonstrate that antibody sample clean-up for intact MS analysis can be achieved without any additional steps required. [file 41592_2019_457_Fig19_ESM.jpg]
