# Supplementary material for: Best practices and benchmarks for intact protein analysis for top-down mass spectrometry
Source: Nat Methods. 2019 Jun 27;16(7):587–94. doi: 10.1038/s41592-019-0457-0 (PMC6719561; doi:10.1038/s41592-019-0457-0)
Supplement: Supplementary file 14 — Supplementary Figs. 1–13, Supplementary Notes 1–5, and Supplementary Protocols 1–5 [file 41592_2019_457_MOESM1_ESM.pdf]

nature methods | SUPPLEMENTARY INFORMATION
https://doi.org/10.1038/s41592-019-0457-0

In the format provided by the authors and unedited.

Best practices and benchmarks for intact protein analysis for top-down mass spectrometry

Daniel P. Donnelly[1,16], Catherine M. Rawlins[1,16], Caroline J. DeHart[2], Luca Fornelli[2], Luis F. Schachner[2], Ziqing Lin[3], Jennifer L. Lippens[4], Krishna C. Aluri[1,5], Richa Sarin[1,6], Bifan Chen[3], Carter Lantz[7], Wonhyeuk Jung[7], Kendall R. Johnson[1], Antonius Koller[1], Jeremy J. Wolff[8], Iain D. G. Campuzano[4], Jared R. Auclair[9], Alexander R. Ivanov[1], Julian P. Whitelegge[10], Ljiljana Paša-Tolić[11], Julia Chamot-Rooke[12], Paul O. Danis[13], Lloyd M. Smith[14], Yury O. Tsybin[15], Joseph A. Loo[7], Ying Ge[3], Neil L. Kelleher[2] and Jeffrey N. Agar[1*]

[1]Barnett Institute of Chemical and Biological Analysis and Departments of Chemistry & Chemical Biology and Pharmaceutical Sciences, Northeastern University, Boston, MA, USA. [2]Departments of Chemistry and Molecular Biosciences and the Proteomics Center of Excellence, Northwestern University, Evanston, IL, USA. [3]Department of Cell and Regenerative Biology, Department of Chemistry, Human Proteomics Program, University of Wisconsin-Madison, Madison, WI, USA. [4]Amgen Research, Discovery Attribute Sciences, Amgen, Thousand Oaks, CA, USA. [5]Alnylam Pharmaceuticals, Cambridge, MA, USA. [6]Biogen, Cambridge, MA, USA. [7]Department of Chemistry and Biochemistry, Department of Biological Chemistry, and UCLA/DOE Institute of Genomics and Proteomics, University of California, Los Angeles, Los Angeles, CA, USA. [8]Bruker Daltonics, Billerica, MA, USA. [9]Biopharmaceutical Analysis Training Laboratory, Northeastern University, Burlington, MA, USA. [10]The Pasarow Mass Spectrometry Laboratory, The Jane and Terry Semel Institute for Neuroscience and Human Behavior, David Geffen School of Medicine, University of California, Los Angeles, Los Angeles, CA, USA. [11]Environmental Molecular Sciences Laboratory, Pacific Northwest National Laboratory, Richland, WA, USA. [12]Mass Spectrometry for Biology Unit, Institut Pasteur, USR 2000, CNRS, Paris, France. [13]Eastwoods Consulting, Boylston, MA, USA. [14]Department of Chemistry, Genome Center of Wisconsin, University of Wisconsin-Madison, Madison, WI, USA. [15]Spectroswiss, Lausanne, Switzerland. [16]These authors contributed equally: Daniel P. Donnelly, Catherine M. Rawlins. *e-mail: j.agar@northeastern.edu

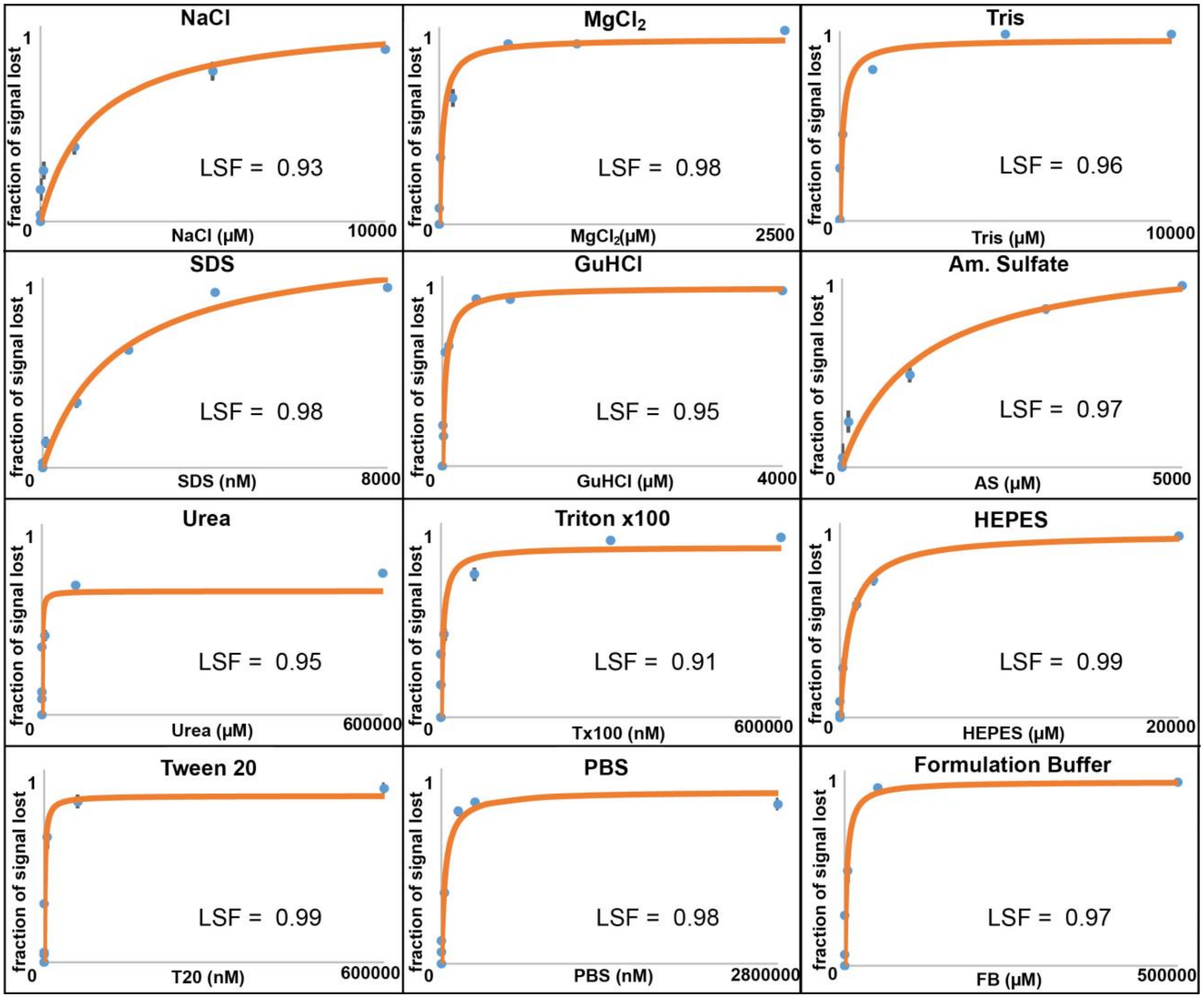

Supplementary Figure 1

Signal suppression curves of common components.

These components are outlined in Figure 1c. The x-axis represents an increasing concentration of interfering substance [C] and the y-axis represents the fraction of signal lost. Each spectrum was collected in triplicate. S/N was calculated as described in the Online Methods. Standard deviation of each data point was calculated and used to produce error bars. The least-squares fitting (LSF) calculation is included to show the quality of fit to the equation.

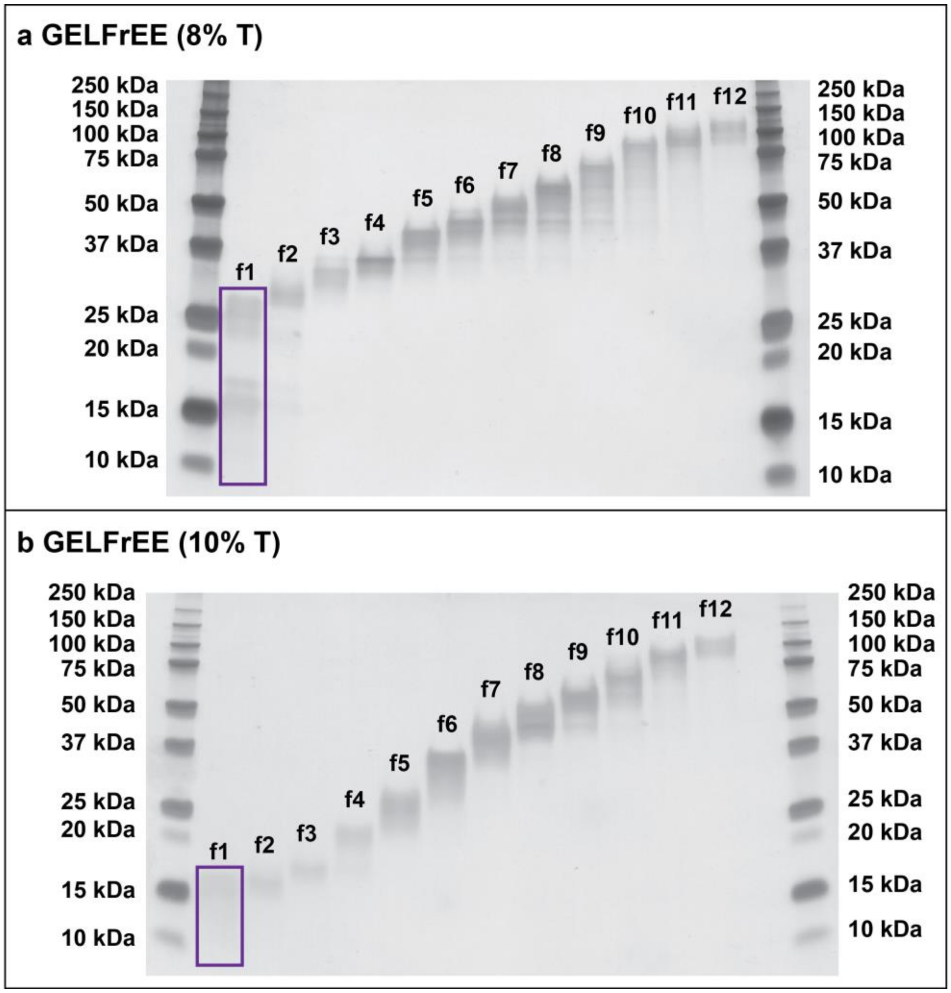

Supplementary Figure 2

Fractionation of human whole-cell lysate prior to top-down mass spectrometry.

Human colorectal cancer cells were lysed and constituent proteins quantified by the methods described by Anderson *et. al*.[1] Aliquots of lysate containing 400 µg were precipitated in acetone, resuspended in 1% SDS containing 50 mM DTT, and resolved on 8% T (a.) or 10% T (b.) gel-eluted liquid fraction entrapment electrophoresis (GELFrEE) cartridges following the respective manufacturer's protocols (GELFrEE 8100 Fractionation System, Expedeon, Inc.). Upon collection of MW-based fractions, 10 µL aliquots were resolved by SDS-PAGE and visualized by $AgNO_3$ stain[2] to gauge protein content and quality of resolution. (a,b) Note that the MW ranges of f1 (purple box) and subsequent fractions differ depending on the GELFrEE cartridge selected. While 8% cartridges (a.) are recommended for quantitative high-throughput top-down MS applications or analysis of higher-MW proteins, 10% (b.) cartridges provide superior resolution in the 5-30 kDa MW range for qualitative high-throughput applications.

1. Anderson, L.C. et al. Identification and Characterization of Human Proteoforms by Top-Down LC-21 Tesla FT-ICR Mass Spectrometry. J Proteome Res 16, 1087-1096 (2017).

2. Shevchenko, A., Wilm, M., Vorm, O. & Mann, M. Mass spectrometric sequencing of proteins silver-stained polyacrylamide gels. Anal Chem 68, 850-858 (1996).

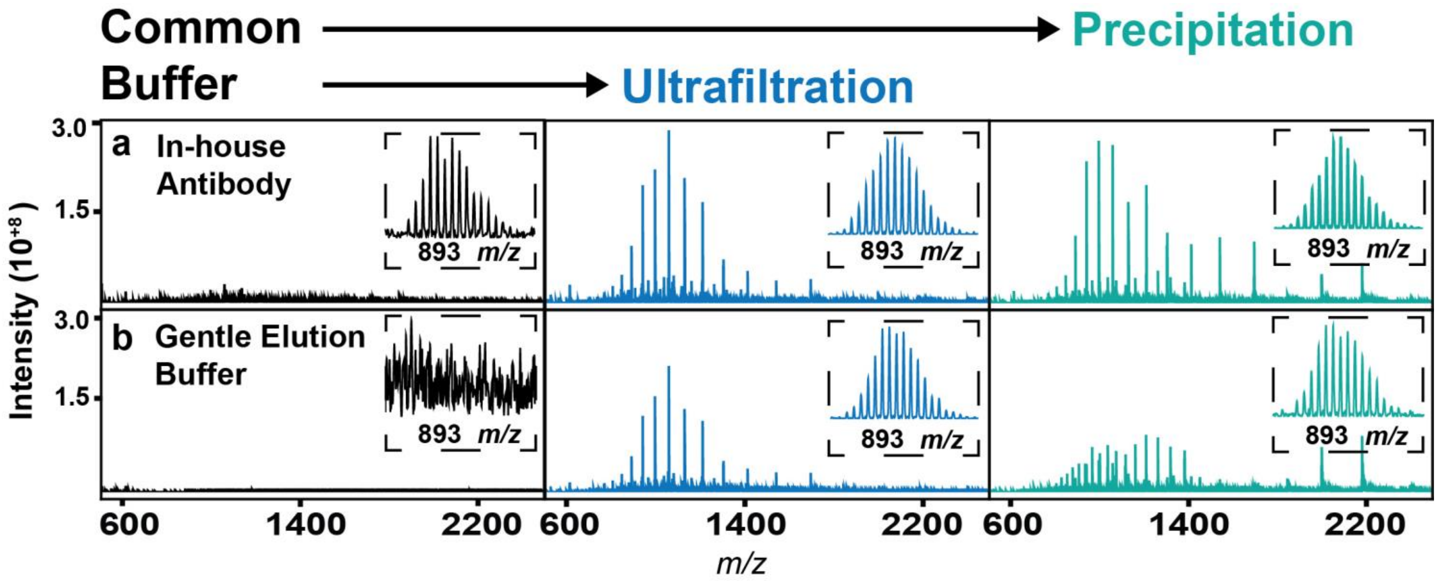

Supplementary Figure 3

Antibody Buffer and Gentle Elution Buffer Ablate MS Signal; MWCO-Ultrafiltration and Precipitation Rescue Signal.

Buffers included (a.) Thermo Gentle Elution Buffer (containing molar salt concentration), and (b.) Antibody buffer (10 mM Arginine, 10 mM Tris HCl, 10 mM histidine, 10 mM potassium phosphate, 10 mM citric acid, pH 5.5). All the above spectra were obtained using a Bruker SolariX FT-ICR mass spectrometer, 9.4T.

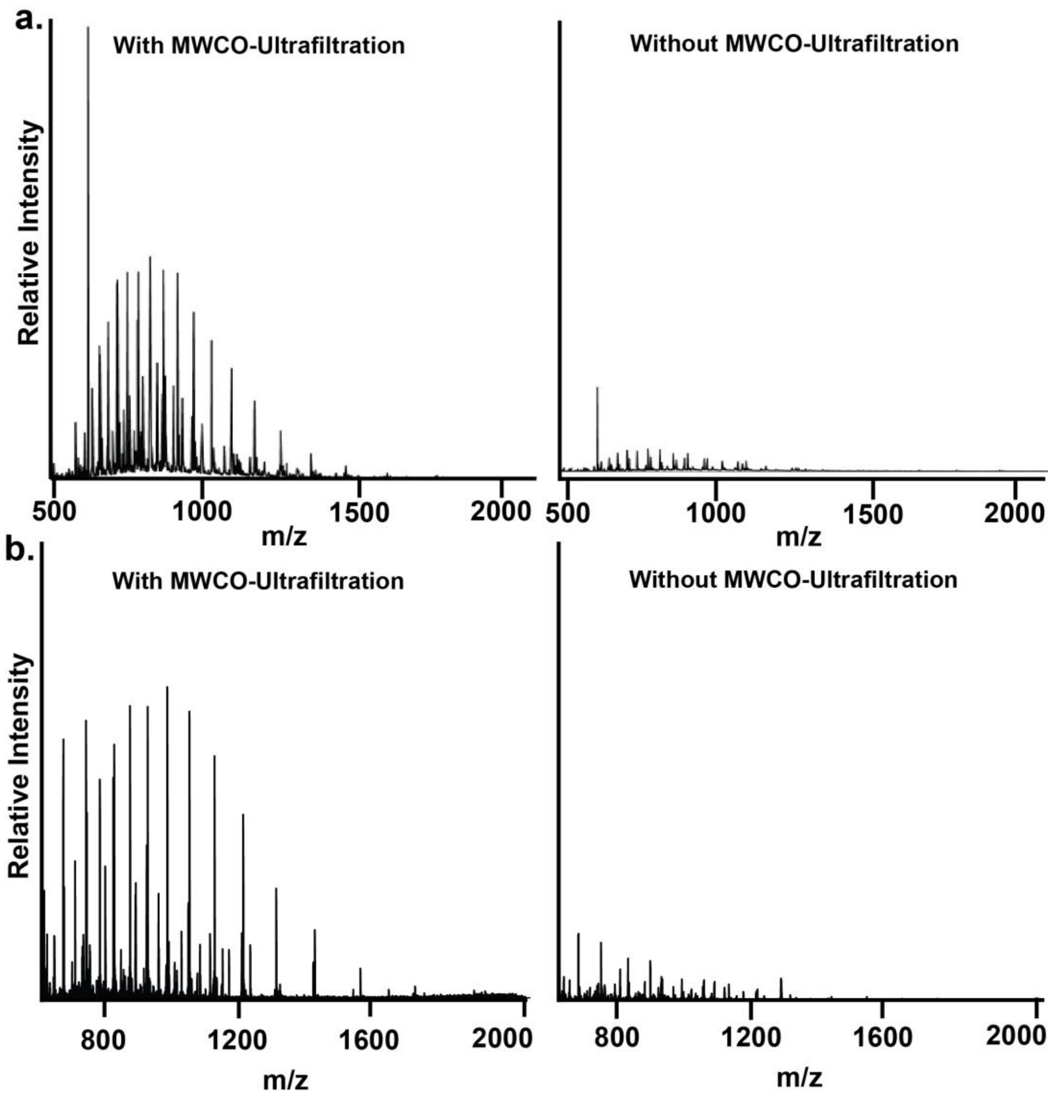

Supplementary Figure 4

Sample Preparation of Protein Mixture following Protocol 3 (MWCO-Ultrafiltration)

These samples were analyzed by direct infusion on a (a.) Waters Xevo G2-S QTOF and a (b.) Thermo Q Exactive Plus.

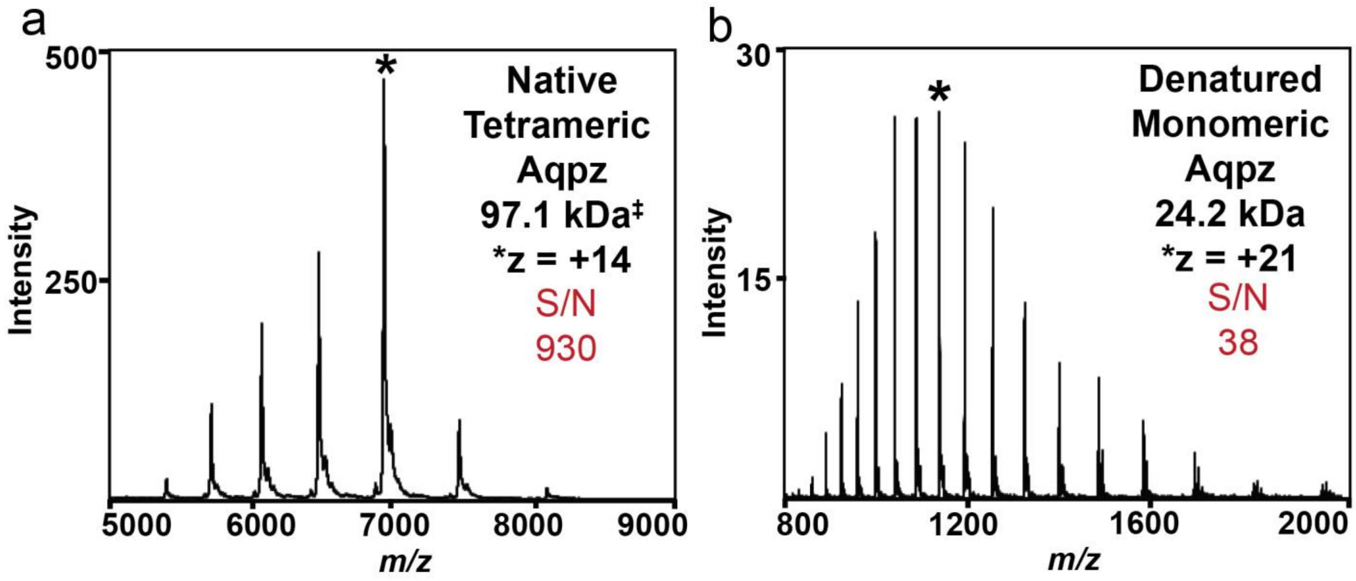

Supplementary Figure 5

Native vs. Denatured MS of AquaporinZ (AqpZ) from E. coli.

Native spectrum was acquired on a Waters Synapt G1 Q-TOF with nanoESI via direct infusion while denatured spectra was acquired on a Waters Synapt G1 Q-TOF via nanoESI-LC-MS. The native sample (a.) and the denatured sample (b.) was acquired at a concentration of 10 µM. *Denotes the most abundant charge state. 24268.7 Da is the deconvoluted mass of the unmodified AqpZ monomer. Formylated AqpZ was also detected with a mass of 24,296.4 Da. ‡Five native tetramer masses were observed corresponding to five unique combinations of formylated and unformylated monomers.[85]

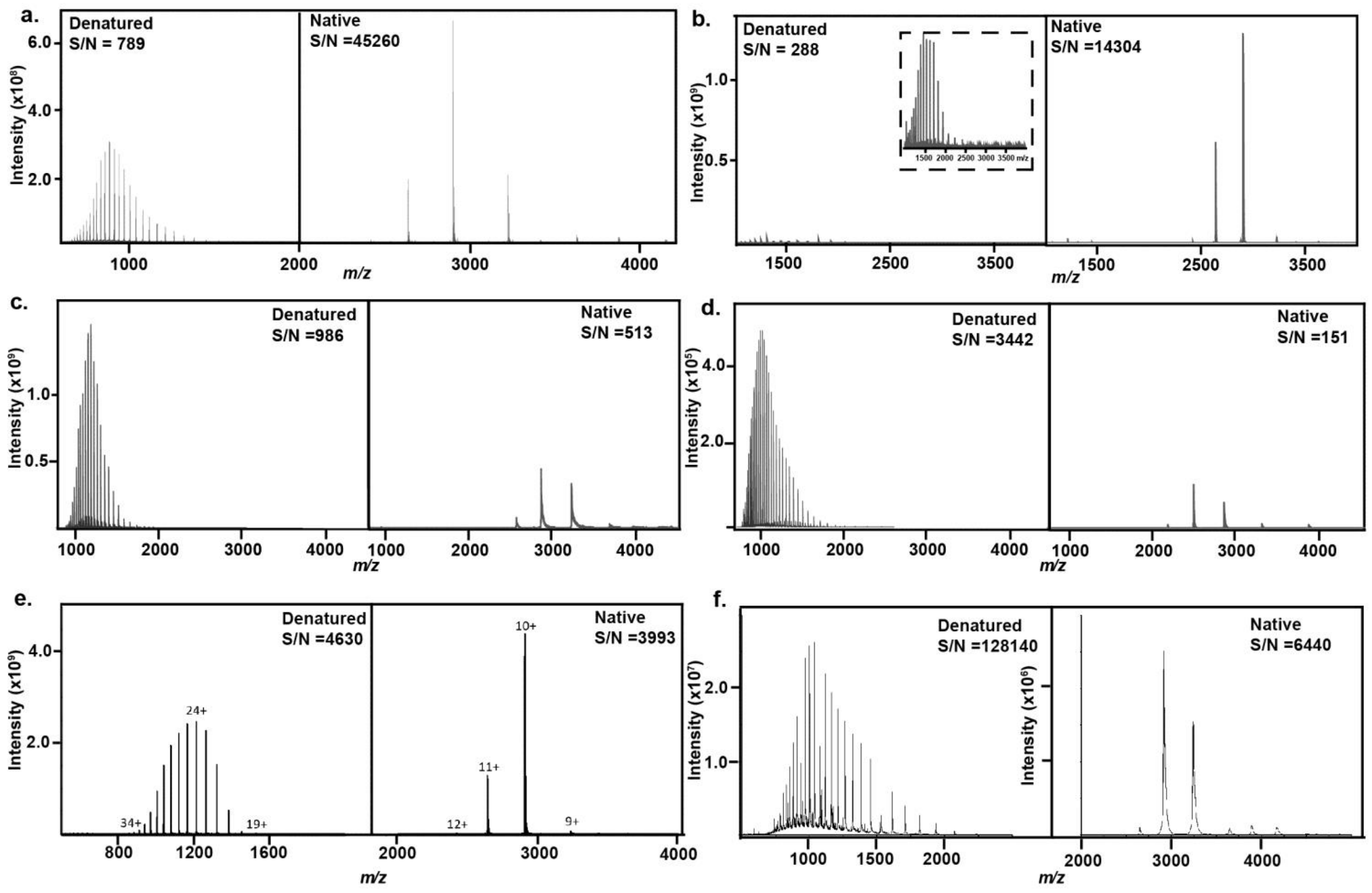

Supplementary Figure 6

Denaturing vs. Native Analysis of Carbonic Anhydrase

Both denaturing and native analysis of carbonic anhydrase was run on a (a.) Thermo Q Exactive HF MS (b.) Bruker 15T SolariX FT-ICR MS, (c.) Bruker 12T SolariX FT-ICR MS, (d.) Bruker maXis II ETD Q-TOF, (e.) Bruker 15T SolariX FT-ICR MS, (f.) Waters Synapt G2Si MS

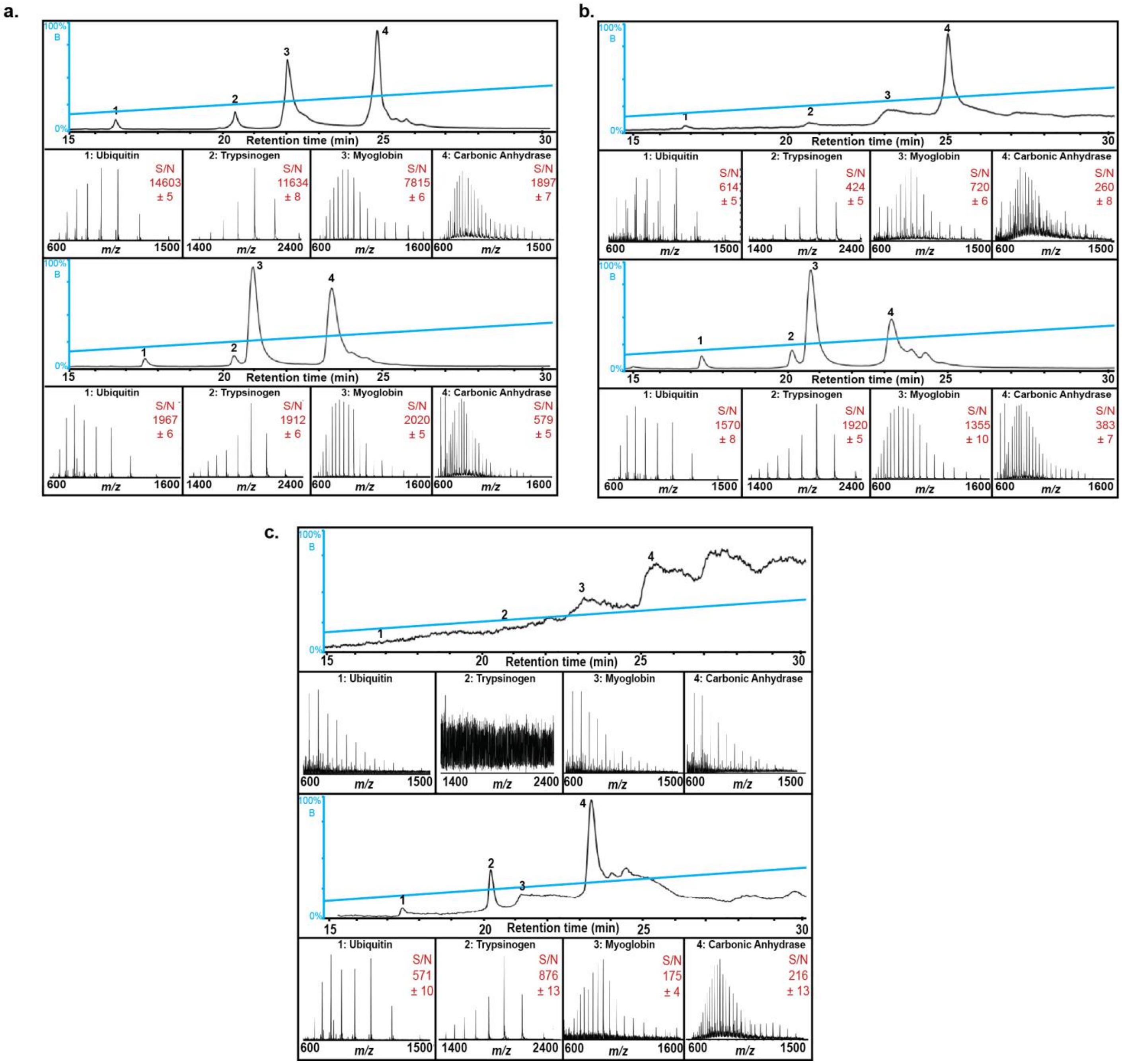

Supplementary Figure 7

LC-MS of protein standard mixture run on Waters Acquity-Xevo G2-S QTOF

Samples were prepared following the given SOP and separated using a PLRP-S (top panel of a, b, and c) or a C4 (bottom panel of a, b, and c) stationary phase. (a.) The final concentrations of each protein loaded onto the column were; 14 pmol ubiquitin, 49 pmol trypsinogen, 109 pmol myoglobin, and 64 pmol carbonic anhydrase. (b.) The final concentrations of each protein loaded onto the column were; 1.4 pmol ubiquitin, 4.9 pmol trypsinogen, 10.9 pmol myoglobin, and 6.4 pmol carbonic anhydrase. (c.) The final concentrations of each protein loaded onto the column were; 0.14 pmol ubiquitin, 0.49 pmol trypsinogen, 1.09 pmol myoglobin, and 0.64 pmol carbonic anhydrase.

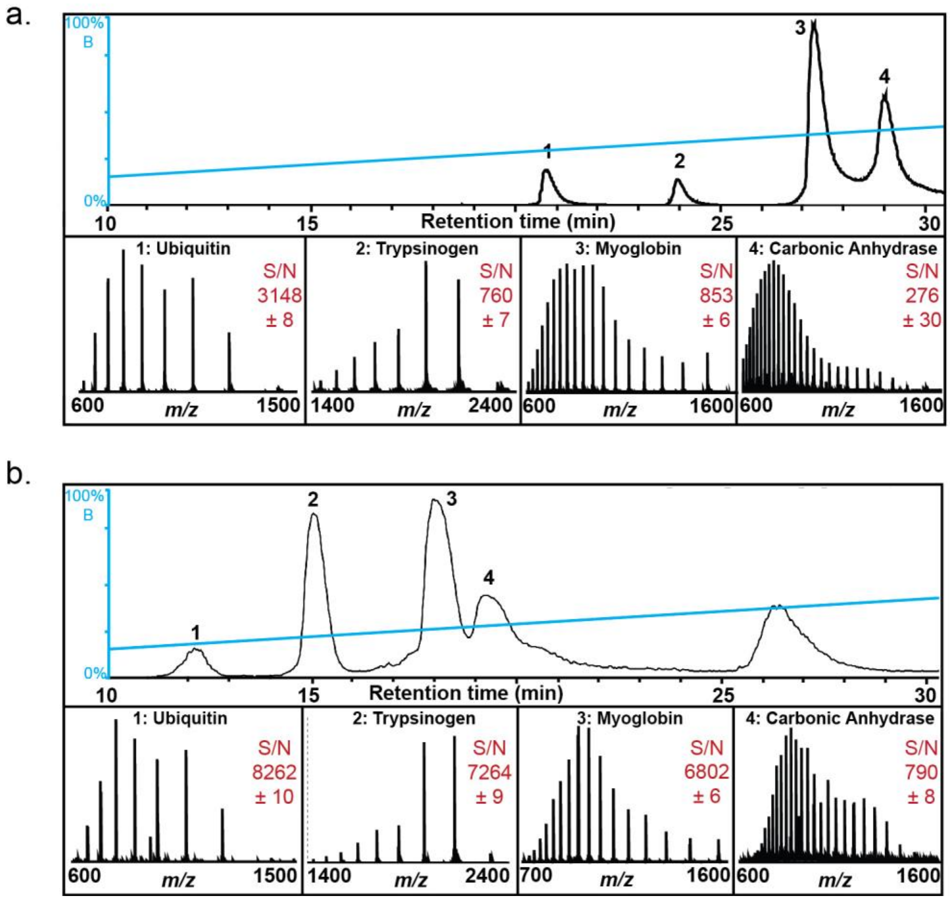

Supplementary Figure 8

LC-MS of protein standard mixture run on Waters nanoAcquity coupled to a Bruker QTOF and a Bruker FT-ICR MS

Samples were prepared following the given SOP and separated using PLRP-S on a Waters nanoAcquity coupled to (a.) a Bruker impact II QTOF and (b.) a Bruker SolariX FT-ICR MS.

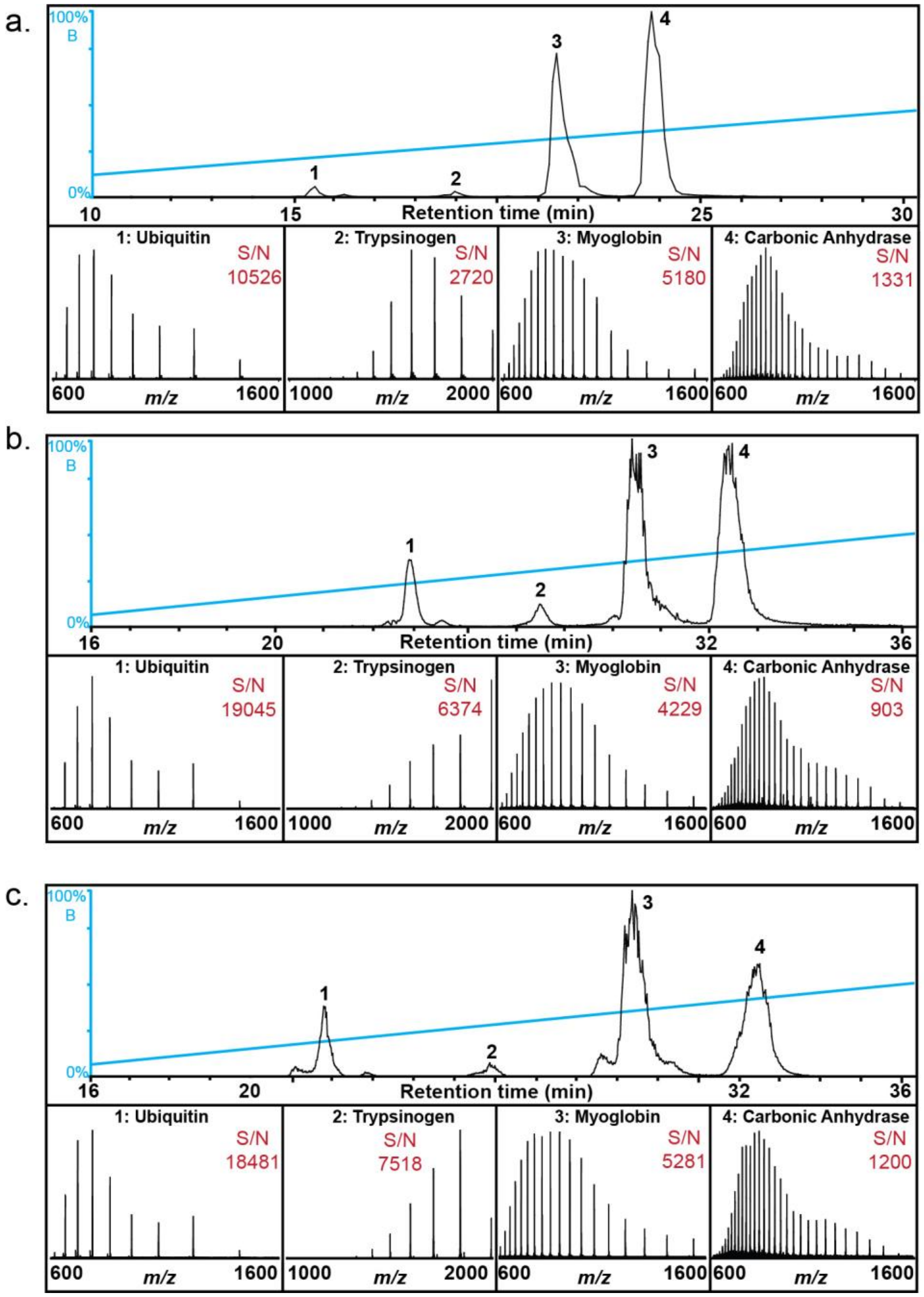

Supplementary Figure 9

LC-MS of protein standard mixture run on a Dionex UPLC coupled to three different orbitrap mass spectrometers

Samples were prepared following the given SOP and separated on a Dionex UPLC coupled to (a.) a Thermo Orbitrap Elite (monolithic stationary phase, (b.) a Thermo Orbitrap Fusion Lumos (PLRP-S stationary phase), (c.) a Thermo Orbitrap QE-HF (PLRP-S stationary phase).

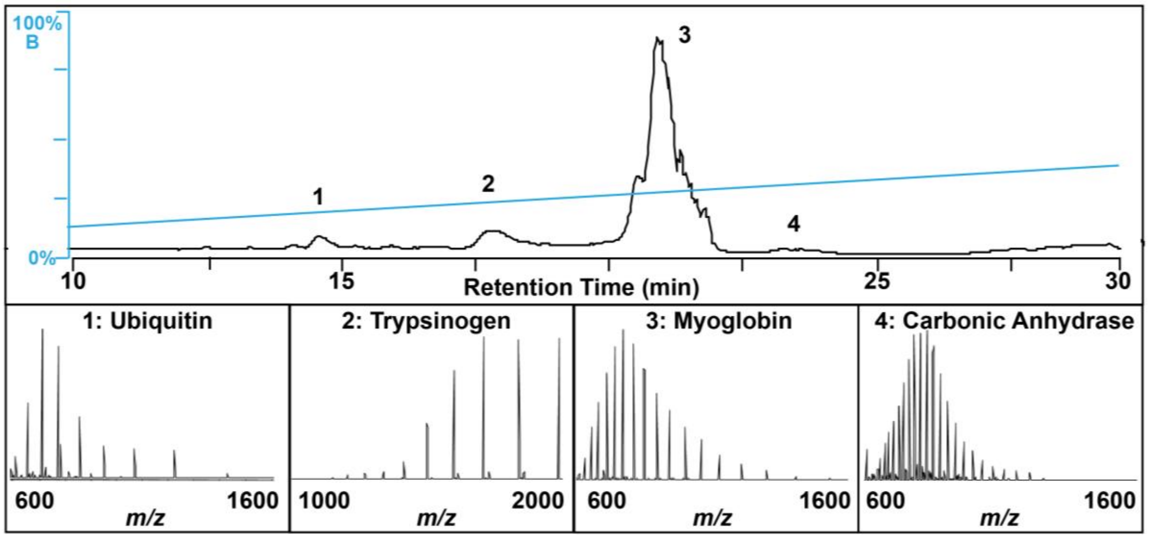

Supplementary Figure 10

LC-MS of protein standard mixture run on a Dionex UPLC coupled to a Thermo Orbitrap Fusion Lumos

Samples were prepared following the given SOP, separated on a Dionex UltiMate 3000 RSLCNano System using PLRP-S stationary phase, and analyzed on a Thermo Fusion Lumos. The final concentrations of each protein loaded onto the column were; 0.14 pmol ubiquitin, 0.49 pmol trypsinogen, 1.09 pmol myoglobin, and 0.64 pmol carbonic anhydrase.

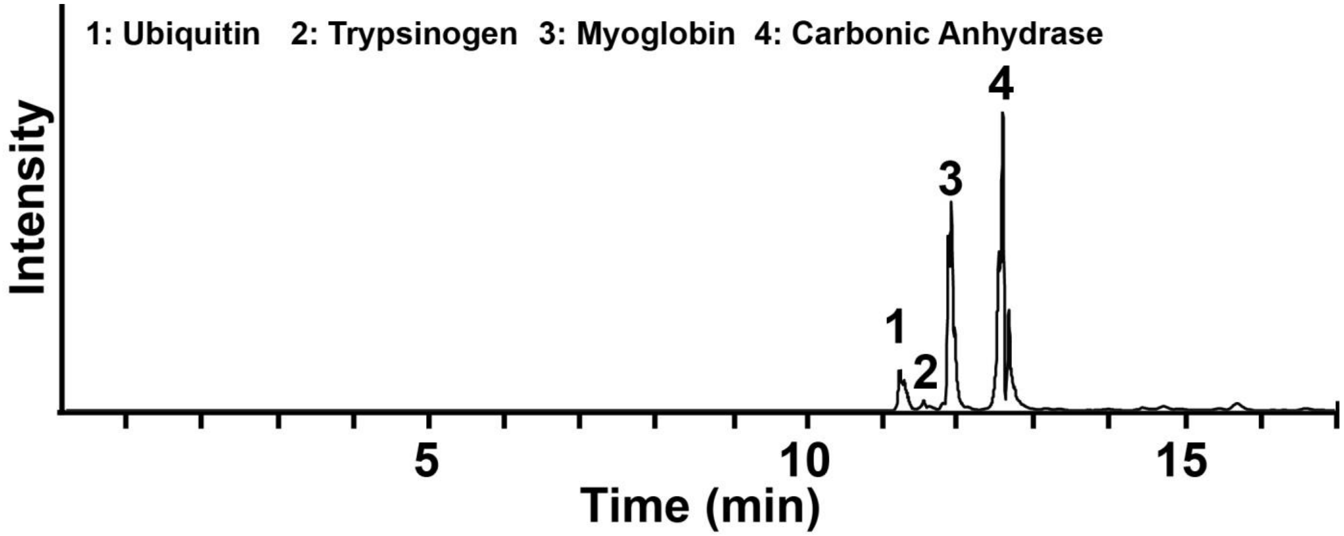

Supplementary Figure 11

Capillary Zone Electrophoresis Separation of Protein Mixture

Separated using a prototype CESI-8000 Plus (AB SCIEX) used with a Neutral OptiMS cartridge.

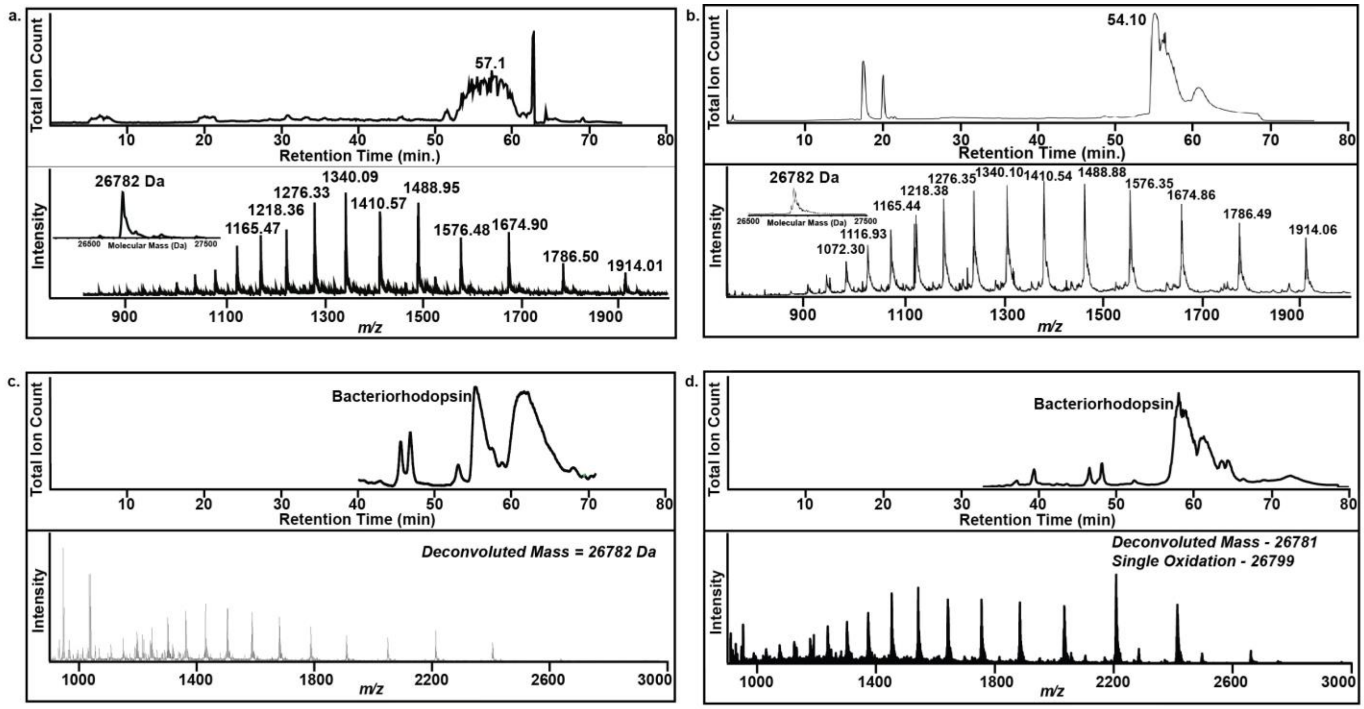

Supplementary Figure 12

LC MS of *Halobacterium salinarum* prepared following Supplemental Protocol 5b

Proteins were separated using a PLRP-S stationary phase (300 Å pore size, 3 µm bead size) and analyzed on a (a.) Waters nanoAcquity interfaced with a Bruker SolariX FT-ICR MS (b.) Waters H-Class Acquity UPLC interfaced with a Waters Xevo G2-S QTOF (c.) Thermo Scientific Vanquish interfaced with a Thermo Orbitrap Q Exactive (d.) Agilent 1290 interfaced with a Thermo Orbitrap Exactive Plus.

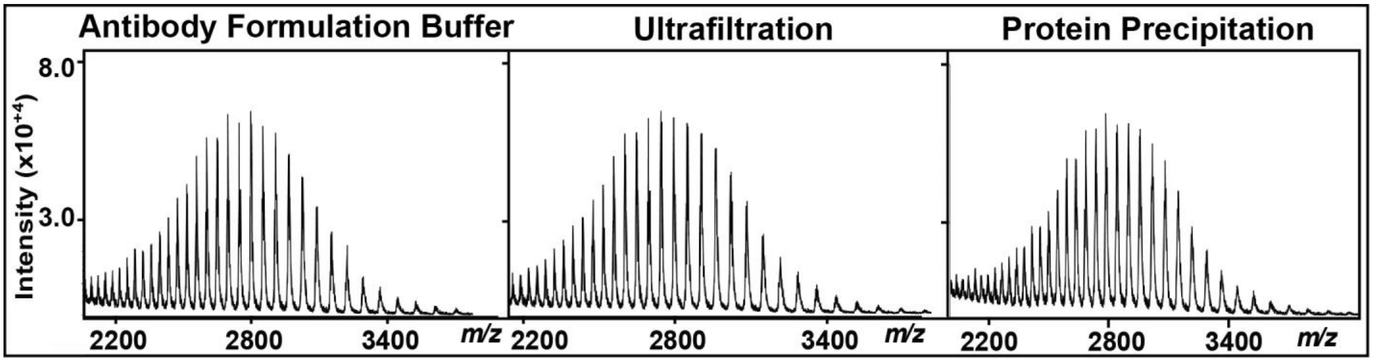

Supplementary Figure 13

LC MS of NIST Antibody on a Waters UPLC-QTOF system using C4 stationary phase.

These results demonstrate that antibody sample clean-up for intact MS analysis can be achieved without any additional steps required.

Supplementary Notes

Supplementary Note 1: Protein Standard Mixture

Bovine ubiquitin (U6253), bovine trypsinogen (T1143), equine myoglobin (M5696), and bovine carbonic anhydrase (C2624) were all purchased from Sigma Aldrich (St. Louis, MO). Stock solutions at 2 mg/mL were made of each solution in HPLC Grade H_2O (Fisher Scientific, Hampton, NH) and the standard was prepared as follows:

Protein	Volume (µL)	Molecular Weight (g/mol)	Stock Concentration (pmol/µL)
**Carbonic Anhydrase**	40	29030	25.7
**Myoglobin**	40	16951	43.9
**Trypsinogen**	25	23981	19.6
**Ubiquitin**	2.5	8560	5.5
**Total**	**107.5**		

This standard mixture was used for the signal suppression curves and for sample clean-up experiments with denaturing mass spectrometry methods. It should be noted that superoxide dismutase (SOD1) can sometimes appear in purchased stocks of bovine carbonic anhydrase. Further details regarding the preparation of this protein standard mixture can be found at http://nrtdp.northwestern.edu/protocols/.

Supplementary Note 2: Details on Signal Suppression Curves

Each interfering substance (listed in **Figure 1c**) was added directly to the concentrated protein standard mixture and then diluted 1:10 in succession. The samples were then diluted 1:40 in 49.95% HPLC grade acetonitrile, 49.95% HPLC grade water, and 0.1% formic acid (v/v), followed by direct infusion. The clean standard mixture, diluted to the same concentration, was used as a

positive control. Three mass spectra were collected from each sample and the S/N was calculated and averaged for the three replicates. Additional samples were run between each data point to calculate the S/N threshold and the equation for the curve. S/N was normalized so that the positive control represented zero loss of S/N. The threshold where 50% of S/N was lost and the least squared fit (LSF) were determined using the sum-of-squares Solver function in Microsoft Excel described below:

$$y_{\%s:n\ lost} = \frac{[C]}{[C] + y_{1/2}}$$

In a similar vein as Michaelis-Menten kinetics, the signal is normalized to 1 (y_{max}) and SC_{50} is, in a sense, the Km; the concentration of [C] at $y_{\%}$ = 0.5 will determine the signal suppression threshold for each buffer component or interfering substance.

Supplementary Note 3: Typical Multicomponent Mixtures of Excipients:

Four "typical buffer" samples made up of commonly used buffers were analyzed for their effects on protein signal. These samples included: 1) 1X PBS (137 mM NaCl, 2.7 mM KCl, 10 mM Na_2HPO_4, 2 mM KH_2PO_4); 2) 50% Thermo RIPA buffer (25mM Tris-HCl pH 7.6, 150mM NaCl, 1% NP-40, 1% sodium deoxycholate, 0.1% SDS); 3) Antibody buffer (10 mM arginine, 10 mM tris-HCl, 10 mM histidine, 10 mM KH_2PO_4, 10 mM citric acid, pH 5.5); and 4) 50% Thermo Fisher Gentle Elution Buffer (containing molar amounts of salts). **Figure 3a-b** shows protein signal in 1X PBS and 50% RIPA, respectively. **Supplementary Figure 3a-b** shows protein signal in antibody buffer and 50% Thermo Fisher Gentle Elution Buffer, respectively.

Supplementary Note 4: Special Considerations for Intact Antibody Mass Spectrometry Additional Details

Humanized IgG1κ (RM8671) was purchased through the National Institute of Standards and Technology (Gaithersburg, MD) The antibody was resuspended in HPLC Grade H_2O to a concentration of 67 µM and stored in 20 µL aliquots at -80 °C. **Figure 3c** spectra were collected on a 9.4 T solariX FTICR MS (Bruker Daltonics, Billerica, MA) with ESI. Each sample was cleaned using Protocols 1-3. The mass range was 610 – 6000 *m/z* with a 1 min 37 s accumulation time (400 scans) and 0.030 s ion accumulation. The time of flight (TOF) was set at 1.500 ms with 20% Sweep Excitation Power and at 64k transient length with $Sine^2$ processing. The flow rate was kept at 2 µL/min with the Dry Gas Temperature at 180 °C and the ESI Capillary voltage at 4.5 kV. The RF Amplitude in the funnel was set to 200 V_{pp} with 150 V for Funnel 1 and 120 V for Skimmer 1. The RF Frequency for the Transfer Optics and the Collision Cell was 2 MHz with the RF Amplitude set to 400 and 1300 V_{pp} respectively.

Supplementary Figure 13 spectra were collected on a H-Class Acquity UPLC system coupled to a Xevo G2-S Q-ToF mass spectrometer (Waters Corp, Milford, MA) using a Acquity UPLC Protein BEH C4 (300 Å pore size, 1.7 µm particle size, 2.1 mm ID x 100 mm) column (Waters Corp, Milford, MA). The solvents were, A: 95% H_2O, 5% acetonitrile, 0.2% formic acid and B: 5% H20, 95% acetonitrile, 0.2% formic acid, and were used with the following gradient:

**Time (min.)**	**% B**
0	5
10	5
12	15
37	55
40	95
43	95
45	5
60	5

The flow rate was 200 µL/min with the capillary voltage set to 3 kV and the sample cone voltage set at 40 V. The source temperature was kept at 150 °C and desolvation temperature 350 °C with a gas flow of 800 L/h. The method ran in the Sensitivity Analyzer mode with a 500 – 4000 *m/z* mass range at 1.00 s/scan time. All analyses and processing were performed using Waters UNIFI 1.7.1 software.

Supplementary Note 5: S/N Calculations

We are not aware of a program with a facile graphical user interface that can preprocess intact protein MS data from multiple vendors. For information on what we consider to be the most accurate method for S/N determination, we invite readers to refer to the work of Tsybin, *et al.*[1] Vendors do not provide preprocessing options that would have allowed us to standardize S/N calculations in this study. In addition, not all vendors offer acceptable deisotoping and deconvolution options for intact proteins, and when they do these options require additional licenses. To avoid subjecting users to the tedious, albeit comprehensive, manual analysis of all charge states, S/N was calculated here by summing the S/N of the top five most abundant charge states for each protein as indicated below. As such, the reported S/N values represent a reproducible subset of the S/N summed from a greater number of charge states during automated deisotoping and deconvolution.

Waters XevoG2: The Waters UNIFI software did not have the capability to calculate protein S/N, therefore, S/N calculations from **Supplementary Figure7** had to be calculated manually. Noise level was estimated from a 10-20 *m/z* segment, adjacent to the most abundant charge state, containing no discernable molecular ion signals. From this segment, a range of intensity that

contained ~97% of signal was estimated. Assuming this contained only normally distributed noise, the range would equal four times the value of "N," *i.e.* N=σ. Signal "S" was defined as the maximum intensity observed within a given charge state. The total S/N across for the five charge states was then summed to give the S/N ratios reported here, and relative standard deviation (RSD) was determined using the three replicates.

Waters Synapt: For **Figure 6**, the S/N for both spectra were calculated by hand. The baseline of the noise was determined as the range of intensity containing no molecular ion signals. Assuming this contained only normally distributed noise, the range would equal two times the value of "N," *i.e.* N=σ. Signal "S" was defined as the maximum intensity observed within a given charge state. The values represented are the sum of the S/N calculations for the top 5 charge states.

Bruker instruments: For **Supplementary Figure 8**, S/N was calculated using DataAnalysis 4.3 (Bruker Daltonics) via the SNAPII (Sophisticated Numerical Annotation Procedure) algorithm with a S/N threshold of 3 (defined as the height of the mass peak above its baseline relative to one standard deviation of the noise) and a quality factor threshold of 0.9. The total S/N across for the five charge states was then summed to give the S/N ratios reported here, and relative standard deviation (RSD) was determined using the three replicates.

Thermo instruments: For **Figures 5**, **Supplementary Figure 6a**, and **Supplementary Figure 9**, the S/N ratios for each protein were calculated manually. For the convenience of users with the

necessary licenses, this method was designed to give results that are comparable to automated deconvolution and deisotoping using the Thermo Xtract or Thrash algorithms. Briefly, the five most abundant charge states of each electrosprayed protein were selected (after averaging all MS1 scans across the respective chromatographic peak). For each charge state peak, the five most abundant isotopomers were considered, and S/N calculated for each isotopomer by the following relation: $\frac{S}{N} = \frac{NL-B}{N-B}$. Here, the signal level is the NL (normalization level) for a given peak, N is empirically determined (and related to the electronic noise level) quantity, and B is the baseline level. The NL, N, and B quantities are returned by Xcalibur 4.0 (Thermo Fisher Scientific). The S/N value for each charge state was calculated by averaging the five single isotopomer S/N values, while the final S/N reported for each protein comprised the sum of the five charge state S/N values from a single analysis.

1. Zhurov, K.O., Kozhinov, A.N., Fornelli, L. & Tsybin, Y.O. Distinguishing analyte from noise components in mass spectra of complex samples: where to cut the noise? *Anal Chem* **86**, 3308-3316 (2014).

Supplementary Protocols

Supplementary Protocol 1: Dilution

In order to sufficiently concentrate protein to allow for dilution out of the typical sample buffers, the protein standard mixture was first precipitated following **Protocol 3**. Pelleted proteins were then resuspended in one tenth of the original volume of sample, to concentrate them to a 10X mixture. The protein mixture in 1X PBS (**Figure 3a**) was diluted 1:50 in 49.95% HPLC grade acetonitrile, 49.95% HPLC grade water, and 0.1% formic acid (v/v). The 10X concentrated sample was diluted 1:500 in 49.95% HPLC grade acetonitrile, 49.95% HPLC grade water, and 0.1% formic acid (v/v). This same procedure was repeated with the RIPA buffer sample (**Figure 3b**).

Supplementary Protocol 2a: MWCO-Ultrafiltration Additional Details

Typical buffer samples were transferred to an Amicon® Ultra 0.5 mL Centrifugal Filter with a regenerated cellulose membrane and a 3 kDa MWCO (Millipore, Billerica, Massachusetts, USA). Samples were diluted with 450 µL of 10 mM ammonium acetate, pH 6.5, and centrifuged at 14000 RPM for 15 min three times (concentrating to 25-50 µL during each centrifugation). Using the same filter devices, samples were exchanged into 2.5 mM ammonium acetate, pH 6.5, by centrifuging at 14000 RPM for 15 minutes three times. Following the last spin, samples were diluted 50-fold their initial volume in 49.95% HPLC grade acetonitrile, 49.95% HPLC grade water, and 0.1% formic acid (v/v). Sample preparation by size exclusion “spin cartridges” can often be a suitable alternative for this protocol.[1]

Supplementary Protocol 2b: Native Membrane Protein Preparation Additional Details

AquaporinZ (AqpZ) from E. coli was provided in 150 mM NaCl, 20 mM Tris-HCl (pH = 8.0), 5% glycerol and 40 mM octyl glucoside. The initial stages of detergent exchange were performed by diluting 150 µg of protein (100 µL) into an equal volume of running buffer containing 2x CMC tetraethylene glycol monooctyl ether (C_8E_4) at RT. This solution was then further buffer and detergent exchanged using FPLC-SEC (GE Healthcare, Sephadex 200, 5/150 GL column). If preparation of membrane proteins for native analysis is performed using MWCO-ultrafiltration instead, we recommend against the use of the same MWCO-ultrafiltration devices used in denaturing experiments (MWCO 3 kDa). Most detergent micelles will not pass through these membranes and are, therefore, concentrated rather than removed. Even after dilution of the sample to below the CMC of a given detergent, the dissociation of non-ionic micelles to monomers occurs slowly enough that equilibrium may not be reached during ultrafiltration. A MWCO membrane with a pore size that exceeds the mass of the empty micelles is recommended.[2, 3] A 50 kDa cutoff membrane, for example, is sufficient to retain protein-micelle complexes of the MS-compatible detergent C_8E_4, but able to remove excess free detergent. We also recommend against using either SEC or MWCO-ultrafiltration for the outright removal of detergents. Removing detergents often results in the loss of native structure, loss of protein solubility, and protein aggregates that clog filters. If outright detergent removal is desired, LC-MS, preceded by precipitation using **Protocol 3** is recommended. If proteins can't be re-solubilized in formic acid following precipitation, LC-MS can be preceded by exchange into MS compatible detergents using the protocol for native MS analysis.

Supplementary Protocol 3: Protein Precipitation Additional Details

All samples were volume normalized prior to precipitation to ensure protein concentration consistency. Each sample was subjected to chloroform/methanol precipitation following a

previously published protocol.[4] 100 µL aliquots of each sample were combined with 400 µL of methanol, 100 µL of chloroform, and 300 µL of HPLC grade H_2O followed by gentle vortexing to ensure complete mixing. Samples were centrifuged for 15 minutes at 14000 RPM and 4 °C in an Eppendorf minispin centrifuge (Hauppauge, New York, USA). The top layer of each sample was aspirated carefully to not disturb the precipitated protein wafer located within the organic/aqueous interface. An additional 400 µL of methanol was added to each sample followed by gentle vortexing and 15 minutes of centrifugation. Solvent was completely removed by aspiration and the pellet was dried briefly. To ensure complete removal of interfering substances, an additional 400 µL of methanol was added to each pellet, followed by vortexing, centrifugation, and aspiration. Following a resolubilization protocol published by Doucette et al.[5], the resulting pellets were chilled at -20 °C for 15 minutes and incubated in 25 µL of 80% formic acid at -20 °C for two minutes. The samples were then mixed and incubated at -20 °C for an additional 15 minutes. 75 µL of cold HPLC grade water was then added to each sample, followed by a final 1:50 dilution in 49.95% HPLC grade acetonitrile, 49.95% HPLC grade water, and 0.1% formic acid (v/v).

Supplementary Protocol 4a: Denaturing Mass Spectrometry Additional Details

All experiments with signal suppression curves and **Protocols 1-3** were performed on a 9.4 T SolariX FT-ICR MS (Bruker Daltonics, Billerica, MA) in positive mode with ESI. The *m/z* range was 154 – 4500 *m/z*, with a 0.1 second ion accumulation, and 41.5 second accumulation time (32 scans). The time of flight (TOF) was set at 1.7 ms with 25% Sweep Excitation Power and at 2 M size resolution. The flow rate was kept at 2 µL/min with the Dry Gas Temperature at 180 °C and the ESI Capillary voltage at 4.5 kV. The RF Amplitude in the funnel was set to 200 V_{pp} with 150 V for Funnel 1 and 40 V for Skimmer 1. The RF Frequency for the Collision Cell was 2 MHz

with the RF Amplitude set to 1300 V_{pp}. The RF Frequency for the Transfer Optics was 4 MHz with the RF Amplitude set to 250 V_{pp}. Although isotopic resolution is not observed here for intact antibodies, it can be achieved with further instrument optimization of the Fourier transform ion cyclotron resonance (FT-ICR) mass spectrometer. Previous studies have combined the use of Continuous Accumulation of Selected Ions (CASI), broadband phase correction, and high field magnets to isotopically resolve intact antibodies.[6-8] Note that nomenclature (*e.g.*, CAD vs. CID vs. HCD) and optimal parameters will vary across different instrumentation platforms.

Supplementary Protocol 4b: Native Mass Spectrometry Additional Details

Native MS has been shown to reduce the signal spreading that is often observed in denaturing MS by producing fewer charge states.[9-11] However, in another study, a 20-fold decrease in S/N was observed in mass spectra obtained from native samples compared to those obtained from denatured samples.[12] Desolvation can be enhanced by increasing collisional activation energy in the source atmosphere/vacuum interface region or collision cell, increasing gas pressure or capillary temperature, and employing nano-ESI. Moreover, small concentrations of supercharging additives (*e.g.*, meta-nitrobenzyl alcohol) that preserve the native conditions of the sample can be used to increase the average charge state of an ionized protein and lower the *m/z* of large proteins into a range compatible with the instrumentation, thereby increasing ion signal in most instruments.[13] Increasing desolvation energy increases the likelihood of bond fragmentation – in particular the loss of labile modifications and non-covalent adducts.

The major application of nMS, for which there are guides[14] and online tutorials (http://msr.dom.wustl.edu/tutorial-native-mass-spectrometry/) available, has been in the targeted study of individual (or simple mixtures of) proteins and protein complexes.[15] Carbonic anhydrase samples analyzed by native mass spectrometry (nMS) were buffer exchanged into 150 mM

ammonium acetate, pH 7.0, following the MWCO-ultrafiltration procedure described in **Protocol 2 & Supplemental Protocol 2**, with Amicon® Ultra 0.5 mL 10-100 kDa MWCO spin filters and a nominal final protein concentration of 10-20 µM. All denaturing vs. native comparative analyses were run using this same sample prep. Ammonium acetate at a concentration of 50-200 mM is the buffer predominantly used in nMS due to its high volatility, low adduction behavior, and non-denaturing properties.[16] By using non-denaturing buffers, non-covalent interactions are preserved and proteins can be maintained in a folded configuration.[16]

Native Analysis Method 1: Bruker 9.4 T SolariX FT-ICR MS

Data were acquired using the Bruker nano-ESI source in positive mode. The capillary was kept at 1200 V with a drying gas temperature of 150 °C. The following source parameters were used: Skimmer 1 at 165 V, Funnel RF amplitude at 250 Vpp, Funnel 1 at 200 V. Time of flight was set to 2.2 ms and sweep excitation was set to 27%. 50 mass spectra were averaged at 4 MW (**Figure 4**).

Native Analysis Method 2: Thermo Fisher Scientific Q Exactive HF MS

A customized Thermo Fisher Scientific Q Exactive HF Mass Spectrometer with Extended Mass Range was utilized for nMS analysis of Carbonic Anhydrase in **Supplementary Figure 6a**. [17] The nMS platform employs direct infusion of sample into a native electrospray ionization (nESI) source held at +2 kV. The instrument was set in EMR MS mode for intact protein mass analysis. The samples were analyzed with the following acquisition parameters: capillary temperature, 330 °C; FT resolution, 15,000 (at 200 *m/z*); S-lens RF level, 50; scan range, 350−10000 *m/z*; desolvation, in-source CID 1-195 V; polarity, positive; microscans, 10; AGC target, 3×10^6;

maximum injection time, 50 ms; averaging, 0; trapping gas pressure setting, 1-4. Mass spectrometric data were analyzed using Thermo Fisher Xcalibur 4.0.

Native Analysis Method 3: Bruker 15 T SolariX FT-ICR MS

Data were acquired using the Bruker Nano-ESI source in positive mode. The capillary was kept at 1100 V with a drying gas temperature of 150 °C. The following source parameters were used: Skimmer 1 at 120 V, Funnel RF amplitude at 250 Vpp, Funnel 1 at 150 V. Time of flight was set to 2.0 ms and sweep excitation was set to 27%. 50 mass spectra were averaged at 1 MW (**Supplementary Figure 6b**).

Native Analysis Method 4: Bruker 12 T SolariX FT-ICR MS

10 µM of carbonic anhydrase in 150 mM ammonium acetate pH=7 was directly infused via a TriVersa NanoMate (Advion Bioscience, Ithaca, NY) nano-ESI source with a spray voltage of 1.8 kV. 50 scans were acquired at 512k words at 202.76-3000 *m/z* with 0.05 s accumulation. The following settings were used: Funnel 1 150 V; Skimmer 1 120 V, Funnel RF amplitude 300 Vpp; Octopole frequency 2 MHz, Octopole RF Amplitude 600 Vpp; Collision cell frequency 1.4 MHz; Collision RF amplitude 2000 Vpp; Transfer Optics time of light 1.5 ms; Transfer Optics frequency 2 MHz; Transfer Optics RF Amplitude 450 Vpp; Sweep excitation power 40%; Gas control 40% (**Supplementary Figure 6c**).

Native Analysis Method 5: Bruker maXis II ETD Q-TOF

10 µM of carbonic anhydrase in 150 mM ammonium acetate pH=7 was directly infused at 3 µL/min via an ESI source. The following settings were used: Mass range 200-6000 *m/z* at 1 Hz acquisition rate; Capillary voltage 3800 V; Nebulizer 1.5 Bar; Dry gas 5 l/min; Dry temp 150 ˚C;

isCID energy 150 eV; Multipole RF 600 Vpp; Collision cell transfer time 110 µs and pre pulse storage 45 µs. 120 scans were averaged (**Supplementary Figure 6d**).

<u>Native Analysis Method 6: Bruker 15 T SolariX FT-ICR MS</u>

Data were acquired using the Bruker nano-ESI source in positive mode. The capillary was kept at 600 V. The following source parameters were used: Skimmer 1 at 25 V, Funnel 1 at 100 V. Time of flight was set to 1.5 ms. 50 mass spectra were averaged (**Supplementary Figure 6e**).

<u>Native Analysis Method 7: Waters SynaptG2Si</u>

120 scans were averages during acquisition. Capillary voltage was kept at 1.6 kV, source temperature at 80 °C, Sample cone at 150 V, Desolvation Temperature at 150 °C, Nanoflow Gas Pressure at 0.3 Bar, and Nebulizer Gas Flow at 6.5 Bar **(Supplementary Figure 6f)**.

<u>Native Analysis Method 8: AqpZ</u>

The nMS of membrane protein AqpZ (E. coli) was acquired on a Waters Synapt G1 QTOF. AqpZ solution was infused into the Q-TOF MS by nanoESI (**Supplementary Figure 5a**). Typical instrument voltages and pressures were: capillary voltage 0.5-1 kV; sample cone 40 V, extraction cone 1V; trap collision energy 4.0 to 110V (range to determine optimal protein ejection voltage) and source backing pressure of 6.0 mbar. The instrument was externally calibrated using a 50 µg/µL CsI solution, over the *m/z* range 500-20,000. In the case of multimeric membrane proteins, care must be taken to balance the removal of detergent ions from the protein-micelle complex and the dissociation of the multimeric complex through increased collisional activation.[18] Too little voltage can render protein charge states unobservable, while too high a voltage can produce lower multimeric species resulting from subunit ejection.[19]

Supplementary Protocol 5a: LC-MS Benchmarks Additional Details

All participating laboratories used the same protein standard mixture and the same SOP which was provided by the National Resource for Translational and Developmental Proteomics (NRTDP) at Northwestern University (http://nrtdp.northwestern.edu/protocols/). All labs used a PLRP-S column; however, as the PLRP-S resin is becoming less commercially available in bulk, some labs also performed the same SOP with their column of choice.

Method 1: Waters UPLC-QTOF

The protein standard mixture was analyzed on an H-Class Acquity UPLC system coupled to a Xevo G2-S Q-TOF mass spectrometer (Waters Corp, Milford, MA). The columns used were an Agilent PLRP-S, 1000 Å pore size, 5 µm particle size, 50 mm bed length, 4.6 mm ID column (Agilent Technologies, Santa Clara, CA) and an Acquity UPLC Protein BEH C4 (300 Å pore size, 1.7 µm particle size, 100 mm bed length, 2.1 mm ID x 100 mm) column (Waters Corp, Milford, MA). The flow rate for the C4 column was 0.2 mL/min while the flow rate for the PLRP-S column was 0.8 mL/min. Solvent A: 95% HPLC Grade H_2O with 0.2% formic acid (Fisher Scientific, Hampton, NH), 5% HPLC Grade Acetonitrile (Fisher Scientific, Hampton, NH), and for Solvent B: 5% HPLC Grade H_2O with 0.2% formic acid, 95% HPLC Grade Acetonitrile. The following gradient was used for both columns:

**Time (min)**	**% B**
0.0	5.0
10.0	5.0
12.0	15.0
37.0	55.0
40.0	95.0
43.0	95.0

45.0	5.0
60.0	5.0

The capillary voltage was set to 3 kV and the sample cone voltage was set at 40 V. The source temperature was kept at 150 °C and desolvation temperature 350 °C with a gas flow of 800 L/h. The method ran in the Sensitivity Analyzer mode with a 500 – 4000 *m/z* mass range. For the PLRP-S column the scan time was 0.5 s, and for the C4 column the scan time was 1 s. **Supplementary Figure 7** shows the results from one of three replicates of the concentrated sample, 1:10 diluted sample, and the 1:100 diluted sample separated with the PLRP-S and C4 stationary phases. For each method, 2.5 µL was injected.

A total ion chromatogram is given in the top panel of each figure, with each chromatographic peak numbered in order of elution. The corresponding mass spectrum for each of the four proteins is presented in four panels below each chromatogram. The undiluted sample (**Supplementary Figure 7a)** contained: 14 pmol ubiquitin, 49 pmol trypsinogen, 109 pmol myoglobin, and 64 pmol carbonic anhydrase. The 1:10 diluted sample (**Supplementary Figure 7b)** contained: 1.4 pmol ubiquitin, 4.9 pmol trypsinogen, 10.9 pmol myoglobin, and 6.4 pmol carbonic anhydrase. The 1:100 diluted sample (**Supplementary Figure 7c)** contained: 0.14 pmol ubiquitin, 0.49 pmol trypsinogen, 1.09 pmol myoglobin, and 0.64 pmol carbonic anhydrase. Unlike the methods that used capillary columns (**Methods 2-6**), the 1:100 dilution showed no protein signal with the PLRP-S (**Supplementary Figure 7c top panel)**. The 1:10 diluted sample produced comparable results within the S/N range of the C4 column (**Supplementary Figure 7b**), but contained broader peaks than expected. The undiluted sample (**Supplementary Figure 7a)** showed ideal chromatographic peaks for both stationary phases.

Method 2: Waters UPLC – Bruker QTOF

The protein standard mixture was prepared as described above except for bovine carbonic anhydrase (PS-121-1) which was purchased from Protea Biosciences (Morgantown, WV). 2 mg/mL stocks of each protein were made in HPLC Grade H_2O (Fisher Scientific, Hampton, NH). This standard mixture was divided into 2 µL aliquots and stored in – 80 °C. One aliquot was diluted to 200 µL and run in triplicate on a nanoAcquity UPLC system (Waters Corp, Milford, MA) coupled to an impact II QTOF (Bruker Daltonics) with a self-packed Agilent PLRP-S (1000 Å pore size, 5 µm particle size), 20 cm bed length, 500 µm ID capillary column. For the LC, Solvent A: 95% HPLC Grade H_2O, 5% HPLC Grade Acetonitrile, 0.2% MS-grade formic acid (Fisher Scientific, Hampton, NH) and Solvent B: 5% HPLC Grade H_2O, 95% HPLC Grade Acetonitrile (Fisher Scientific, Hampton, NH), 0.2% MS-grade formic acid were used with the following gradient:

Time (min)	% B
0.0	5.0
5.0	5.0
42.0	60.0
44.0	95.0
46.0	95.0
47.0	5.0
60.0	5.0

For each replicate, 5 µL was injected at a flow rate of 12 µL/min with the mass range at 500 – 2500 *m/z* for a final injected amount of 0.14 pmol ubiquitin, 0.49 pmol trypsinogen, 1.09 pmol myoglobin, and 0.64 pmol carbonic anhydrase. The Collision Cell RF was set to 2500 Vpp, the

Capillary voltage at 4500 V, the Funnel RF at 400 V_{pp}, the Collision cell energy at 8 V, the Quadrupole energy at 4 V, and the in-source CID at 40 V. The source gas temperature was set to 220 °C with a 4 L/min flow rate. The results are displayed in **Supplementary Figure 8a** with the S/N calculated for each protein.

<u>Method 3: Waters UPLC – Bruker FTICR</u>

All sample preparation, column, and LC parameters were identical to those described above in **Method 2**. In this method, the nanoAdvance HPLC (Bruker Daltonics, Billerica, MA) was coupled to a solariX XR 12T FTICR (Bruker Daltonics, Billerica, MA) with a capillary column of 15 cm bed length, 250 µm ID, running at 4 µL/min. The mass range was 500 – 2500 *m/z* with a 0.08 s ion accumulation with 1.4 s per scan. The TOF was set at 1.00 ms with 30% Sweep Excitation Power and at 1M transient length with Full Sine processing. The dry gas temperature was set to 220 °C with a 4 L/min flow rate and the ESI Capillary voltage at 4.5 kV. The RF Amplitude in the funnel was set to 150 V_{pp} and 40 V for in-source CID. The RF Frequency for the Transfer Optics was 4 MHz with 350 V_{pp} RF Amplitude and the Collision Cell was 2 MHz with the RF Amplitude set to 2000 V_{pp}. The results are displayed in **Supplementary Figure 8b** with the S/N calculated for each protein.

<u>Method 4: Dionex UPLC – Thermo Orbitrap Elite</u>

The intact protein standard mixture was prepared as described above and divided into 2.5 µL aliquots for storage at -80 °C. Prior to analysis, aliquots of standard were diluted 1:240 in 600 µL Solvent A (95% Optima grade water, 5% Optima grade acetonitrile, and 0.2% MS-grade formic acid; all Fisher Scientific, Hampton, NH), for a final injected amount of 0.14 pmol ubiquitin, 0.49 pmol trypsinogen, 1.09 pmol myoglobin, and 0.64 pmol carbonic anhydrase. Ultra-high

performance liquid chromatography (UPLC) was performed on-line with the MS using a Dionex Ultimate 3000 (Thermo Fisher Scientific, San Jose, CA). Trap (150 µm ID x 2 cm) and analytical (75 µm ID x 15 cm) columns were packed in-house with PLRP-S resin (5 µm, 1000 Å) (Agilent Technologies, Santa Clara, CA) and enclosed within a column oven compartment maintained at 45 °C. Monolithic PepSwift trap (150 µm ID x 0.5 cm) and ProSwift RP-4H analytical (100 µm ID x 50 cm) columns were purchased from Thermo Fisher Scientific, conditioned according to the manufacturer's protocol, and enclosed within a column oven compartment maintained at 35 °C. 6 µL injections were concentrated and desalted on the trap column in Solvent A for 3 minutes at 10 µL/min (Monolithic) or 10 minutes at 3 µL/min (PLRP-S). Intact proteins were then further resolved prior to MS analysis using the gradients of Solvent A and Solvent B (5% Optima grade water, 95% Optima grade acetonitrile, and 0.2% MS-grade formic acid) shown in the tables below:

Monolithic: 1 µL/min flow rate

Time (min)	% B
0.0	5.0
3.0	5.0
33.0	50.0
35.0	95.0
38.0	95.0
39.0	5.0
53.0	5.0

PLRP-S: 0.3 µL/min flow rate

Time (min)	% B
0.0	5.0
10	5.0
12.0	15.0
37.0	55.0
40.0	95.0
43.0	95.0

45.0	5.0
60.0	5.0

Chromatographically resolved proteins were introduced into the MS using a custom nano-ESI source containing a high-voltage union (Thermo Fisher Scientific) coupled to the end of the analytical column, through which a 1.9-2.2 kV potential was applied, and connected to a 15 µm ID nanospray emitter (New Objective, Woburn, MA) self-packed with 2 mm of PLRP-S resin.

Intact protein (MS1) spectra were acquired over an *m/z* range of 500-2000 at an FT resolution of 120,000 (at 400 *m/z*) on an LTQ-Velos Orbitrap Elite mass spectrometer (Thermo Fisher Scientific) operating in "protein mode" under control of Xcalibur (Thermo Fisher Scientific). MS1 method details included 4 microscans, an AGC target of 1 x 10^6 ions, a maximum injection time of 1s, and an additional 15 V applied within the source region to facilitate desolvation. Source region parameters also included a transfer capillary temperature of 320 °C and an S-lens RF amplitude of 50%. Mass spectrometric data were analyzed using Xcalibur 4.0 (Thermo Fisher Scientific). The resulting chromatogram, intact protein spectra, and respective S/N calculation from a representative replicate injection are shown in **Supplementary Figure 9a**.

Method 5: Dionex UPLC – Thermo Orbitrap Fusion Lumos

All sample preparation, PLRP-S column, and LC parameters were identical to those described above in **Method 4**. Intact protein (MS1) spectra were acquired over an *m/z* range of 400-2000 at an FT resolution of 120,000 (at 200 *m/z*) on an Orbitrap Fusion Lumos mass spectrometer (Thermo Fisher Scientific) operating in "intact protein mode" under control of Xcalibur. MS1 method details included 4 microscans, an AGC target of 2 x 10^5 ions, a maximum ion injection time of 100 ms, and an additional 15V applied within the source region to facilitate desolvation. Source region

parameters also included a transfer capillary temperature of 320 °C and an ion funnel RF amplitude of 30%. Mass spectrometric data were analyzed using Xcalibur 4.0 (Thermo Fisher Scientific). The resulting chromatogram, intact protein spectra, and respective S/N calculations from a representative injection are shown in **Supplementary Figure 9b**.

<u>Method 6: Dionex UPLC – Thermo QE-HF</u>

All sample preparation, PLRP-S column, and LC parameters were identical to those described above in **Method 4**. Intact protein (MS1) spectra were acquired over an *m/z* range of 500-2000 at an FT resolution of 120,000 (at 200 *m/z*) on a QE-HF mass spectrometer (Thermo Fisher Scientific) operating in "intact protein mode" under control of Xcalibur. MS1 method details included 4 microscans, an AGC target of 1 x 10^6 ions, a maximum ion injection time of 50 ms, and an additional 15V applied within the source region to facilitate desolvation. Source region parameters also included a transfer capillary temperature of 320 °C and an S-lens RF amplitude of 50%. Mass spectrometric data were analyzed using Xcalibur 4.0 (Thermo Fisher Scientific). The resulting chromatogram, intact protein spectra, and respective S/N calculations from a representative injection are shown in **Supplementary Figure 9c**.

<u>Method 7: Dionex UltimMate 300 RSLCnano System – Thermo Fusion Lumos</u>

The Protein standard mixture was prepared as described in the given SOP by diluting 14 pmol ubiquitin, 49 pmol trypsinogen, 109 pmol myoglobin, and 64 pmol carbonic anhydrase in 100 uL of 95% ACN, 5% Water, 0.2% formic acid and injecting 1 µL for analysis. Samples were separated on an in-house pulled 100 µm ID capillary with integrated spray tip packed with PLRP-S resin (1000 Å pore size, 5 µm particle size) to 15 cm. Sample was directly loaded onto this column by the autosampler at 0.3 ul/min for 30 min and the same gradient as described in **Method 4** was

applied. The EASY-spray source (Thermo Fisher Scientific) was used in conjunction with a Jailbreak Column Heater (Phoenix S&T) to heat to 50 °C and position the column to the front of the mass spectrometer. Intact protein (MS1) spectra were acquired over an *m/z* range of 500-2000 at an FT resolution of 75,000 (at 400 *m/z*) on a Thermo Orbitrap Fusion Lumos (Thermo Fisher Scientific) operating in "protein mode" under control of Xcalibur (Thermo Fisher Scientific). MS1 method details included 4 microscans, an AGC target of 1 x 10^6 ions, a maximum injection time of 50ms, and an additional 15V applied within the source region to facilitate desolvation. Source region parameters also included a transfer capillary temperature of 320 °C and an S-lens RF amplitude of 60%. Mass spectrometric data were analyzed using Xcalibur 4.0 (Thermo Fisher Scientific). The resulting chromatogram, intact protein spectra, and respective S/N calculation from a representative replicate injection are shown in **Supplementary Figure 10.**

Supplementary Protocol 5b: Denaturing Reversed Phase LC-MS of bacteriorhodopsin from *Halobacterium salinarum*

<u>Method 1: Agilent 1200 HPLC – Thermo LTQ (Figure 6)</u>

Bacteriorhodopsin from *Halobacterium salinarum* was purchased through Sigma-Aldrich (B0184). 1 mg of protein was suspended in 100 µL of 1% CHAPS in HPLC-grade water to a final concentration of 10 mg/mL. Membrane protein solution was aliquoted into 25 µg aliquots and stored at 4 °C until use (<5 days) or -80 °C for extended storage. Prior to analysis, 1 aliquot of protein (25 µg) was mixed with 10 µL of 88% formic acid (ACS Grade). Upon addition of acid, the protein solution changed from purple to orange, and was immediately injected onto the column (approximately 1-3 minutes depending on plumbing). The idea was to minimize exposure to formic acid (less than 2 mins) as it is known to modify proteins at this concentration. 3 µL (224 pmol) of protein is injected onto a PLRP-S (Agilent) reversed-phase column (300 Å

pore size, 3 µm bead size). Solvent A was water + 0.1% formic acid and solvent B was 49.95% HPLC grade acetonitrile, 49.95% HPLC grade isopropanol, and 0.1% formic acid. We used an increasing gradient of solvent B during separation (specified below) and a flow rate of 0.120 mL/min:

**Time (min)**	**% Solvent B**
0	5
5	5
10	30
50	80
55	99
65	99
66	5
75	5

Method 2: Waters nanoAcquity – Bruker SolariX FT-ICR (Supplementary Figure 12a)

25 µg of bacteriorhodopsin from Halobacterium salinarum was suspended in 2.5 µL of HPLC grade water to a final concentration of 10 mg/mL. Water was used instead of 1% CHAPS due the FT-ICR's sensitivity to CHAPS, which overpowered protein signal. 110 µL of 88% formic acid was added to the protein solution, turning it from a deep purple to an orange immediately. 3 µL (22.4 pmol) of sample was loaded onto a self-packed PLRP-S capillary column (7 cm length, 100 µm I.D., 300 Å pore size, 3 µm bead size). The same gradient given above was run at 2 µL/min.

Mass analysis was performed on a 9.4 T SolariX FT-ICR-MS (Bruker Daltonics, Billerica, MA) in the positive mode with ESI. The *m/z* range was 154 – 5000 *m/z*, with a 0.15 second ion accumulation, The time of flight (TOF) was set at 1.4 ms with 18% Sweep Excitation Power and

at 1M size resolution. Dry Gas Temperature was kept at 180 °C and the ESI Capillary voltage at 4.5 kV. The RF Amplitude in the funnel was set to 200 V_{pp} with 150 V for Funnel 1 and 40 V for Skimmer 1. The RF Frequency for the Collision Cell was 2 MHz with the RF Amplitude set to 1300 V_{pp}. The RF Frequency for the Transfer Optics was 4 MHz with the RF Amplitude set to 250 V_{pp}.

Method 3: H-Class Acquity UPLC - Xevo G2-S Q-TOF (Supplementary Figure 12b)

Bacteriorhodopsin from *Halobacterium salinarum* was prepared following the same procedure as described in **Method 1**, above. 3 uL (224 pmol) of protein was loaded onto an PLRP-S column (3 µm, 300 Å, 2.1x50 mm) (Agilent Technologies, Santa Clara, CA). Samples were run at 200 µL/min. Solvent A was 100% HPLC Grade H_2O with 0.1% formic acid and Solvent B was 49.95% HPLC Grade Acetonitrile, 49.95% Isopropanol, 0.1% formic acid. The same linear gradient applied in B was used. The capillary voltage was set to 3 kV and the sample cone voltage was set at 40 V. The source temperature was kept at 150 °C and desolvation temperature at 350 °C with a gas flow of 800 L/h. The method ran in the Sensitivity Analyzer mode with a 500 – 4000 *m/z* mass range. Scan time was set to 1 s.

Method 4: Thermo Scientific Vanquish – Thermo Q Exactive (Supplementary Figure 12c)

Bacteriorhodopsin from *Halobacterium salinarum* was prepared following the same procedure as described in **Method 1** above. 3 uL (224 pmol) of protein was loaded onto an PLRP-S column (3 µm, 300 Å, 2.1x50 mm) (Agilent Technologies, Santa Clara, CA). The following linear gradient was used at a flow rate of 400 µL/min.

**Time (min)**	**% B**

0.0	5.0
5	5.0
80	100

The mass spectrometer was run in positive ion mode at 140,000 resolution, scanning a range *m/z* from 850-3000 *m/z*. Mass spectra were collected after 40 minutes to ensure CHAPS was directed towards waste. Capillary temperature was kept at 263 °C and S-Lens RF level was set at 50. In-source CID was set at 5.0 eV.

Method 5: Agilent 1290 – Thermo Exactive Plus (Supplementary Figure 12d)

Bacteriorhodopsin from Halobacterium salinarum was prepared following the same procedure as described in **Method 1** above. 3 uL 224 (pmol) of protein was loaded onto an PLRP-S column (3 µm, 300 Å, 2.1x50 mm) (Agilent Technologies, Santa Clara, CA). The following linear gradient was run at a flow rate of 0.3 mL/min:

**Time (min)**	**% B**
0.0	5.0
5	5.0
80	100

The mass spectrometer was run in positive ion mode at 140,000 resolution, scanning a range *m/z* from 900-3500 *m/z*. Capillary temperature was kept at 265 °C and S-Lens RF level was set at 60. In-source CID was set at 20.0 eV.

Denaturing LC-MS of Membrane Protein AqpZ

LC-MS of AqpZ (E. coli) was performed using an Acquity UPLC, XevoQ-ToF (Waters Corporation, MS Technologies Center) and an Agilent ZORBAX RRHD 300SB-C3, 2.1 x 50 mm, 1.8 µm, 300 Å column. Using mobile phases H_2O containing trifluoroacetic acid, 0.1% v/v, formic acid, 0.1% v/v, as mobile phase A and 90% n-propanol containing trifluoroacetic acid, 0.1% v/v, formic acid, 0.1% v/v, as mobile phase B. UPLC analysis was achieved using a 5 minute UPLC method (including re-equilibration) operating at a flow rate of 400 µL/min and a column temperature of 65 °C. AqpZ analyzed, as received from purification, under the above UPLC-MS conditions yielded spectra displayed in **Supplementary Figure 5b**. Contrary to nMS, denaturing LC-MS requires no further sample preparation (FPLC detergent exchange and buffer exchange) prior to analysis. The instrument was externally calibrated using a 50 µg/µL CsI solution over m/z range of 1000-4500. Real-time lock mass correction was performed on the leucine encephalin dimer (m/z 1111.5464).

1. Porath, J. & Flodin, P. Gel filtration: a method for desalting and group separation. *Nature* **183**, 1657-1659 (1959).
2. Laganowsky, A., Reading, E., Hopper, J.T.S. & Robinson, C.V. Mass Spectrometry of Intact Membrane Protein Complexes. *Nature protocols* **8**, 639-651 (2013).
3. Laganowsky, A. et al. Membrane proteins bind lipids selectively to modulate their structure and function. *Nature* **510**, 172-175 (2014).
4. Wessel, D. & Flügge, U.I. A method for the quantitative recovery of protein in dilute solution in the presence of detergents and lipids. *Analytical Biochemistry* **138**, 141-143 (1984).
5. Doucette, A.A., Vieira, D.B., Orton, D.J. & Wall, M.J. Resolubilization of precipitated intact membrane proteins with cold formic acid for analysis by mass spectrometry. *Journal of proteome research* **13**, 6001-6012 (2014).
6. Valeja, S.G. et al. Unit Mass Baseline Resolution for an Intact 148 kDa Therapeutic Monoclonal Antibody by FT-ICR Mass Spectrometry. *Analytical chemistry* **83**, 8391-8395 (2011).
7. Li, H., Wolff, J.J., Van Orden, S.L. & Loo, J.A. Native Top-Down Electrospray Ionization-Mass Spectrometry of 158 kDa Protein Complex by High-Resolution Fourier Transform Ion Cyclotron Resonance Mass Spectrometry. *Analytical Chemistry* **86**, 317-320 (2014).
8. Shaw, J.B. & Brodbelt, J.S. Extending the isotopically resolved mass range of Orbitrap mass spectrometers. *Anal Chem* **85**, 8313-8318 (2013).
9. de la Mora, J.F. Electrospray ionization of large multiply charged species proceeds via Dole's charged residue mechanism. *Anal Chim Acta* **406**, 93-104 (2000).

10. Compton, P.D., Zamdborg, L., Thomas, P.M. & Kelleher, N.L. On the Scalability and Requirements of Whole Protein Mass Spectrometry. *Analytical Chemistry* **83**, 6868-6874 (2011).
11. Belov, A.M. et al. Analysis of Proteins, Protein Complexes, and Organellar Proteomes Using Sheathless Capillary Zone Electrophoresis - Native Mass Spectrometry. *Journal of The American Society for Mass Spectrometry* **28**, 2614-2634 (2017).
12. Konermann, L., Ahadi, E., Rodriguez, A.D. & Vahidi, S. Unraveling the Mechanism of Electrospray Ionization. *Analytical Chemistry* **85**, 2-9 (2013).
13. Iavarone, A.T. & Williams, E.R. Mechanism of charging and supercharging molecules in electrospray ionization. *J Am Chem Soc* **125**, 2319-2327 (2003).
14. Kirshenbaum, N., Michaelevski, I. & Sharon, M. Analyzing large protein complexes by structural mass spectrometry. *Journal of visualized experiments : JoVE* (2010).
15. Heck, A.J.R. Native mass spectrometry: a bridge between interactomics and structural biology. *Nat Methods* **5**, 927-933 (2008).
16. Loo, J.A. Studying noncovalent protein complexes by electrospray ionization mass spectrometry. *Mass Spectrometry Reviews* **16**, 1-23 (1997).
17. Belov, M.E. et al. From protein complexes to subunit backbone fragments: a multi-stage approach to native mass spectrometry. *Anal Chem* **85**, 11163-11173 (2013).
18. Campuzano, I.D.G. et al. Native MS Analysis of Bacteriorhodopsin and an Empty Nanodisc by Orthogonal Acceleration Time-of-Flight, Orbitrap and Ion Cyclotron Resonance. *Analytical Chemistry* **88**, 12427-12436 (2016).
19. Lippens, J.L. et al. Fourier Transform-Ion Cyclotron Resonance Mass Spectrometry as a Platform for Characterizing Multimeric Membrane Protein Complexes. *Journal of The American Society for Mass Spectrometry* (2017).
20. Zhurov, K.O., Kozhinov, A.N., Fornelli, L. & Tsybin, Y.O. Distinguishing analyte from noise components in mass spectra of complex samples: where to cut the noise? *Anal Chem* **86**, 3308-3316 (2014).
